# Female researchers are under-represented in the Colombian science infrastructure

**DOI:** 10.1371/journal.pone.0298964

**Published:** 2024-03-06

**Authors:** Andrea Paz, Carolina Pardo-Díaz

**Affiliations:** 1 Department of Environmental Systems Science, Institute of Integrative Biology, ETH Zürich, Zürich, Switzerland; 2 Département de Sciences Biologiques, Université de Montréal, Montréal, Canada; 3 Department of Biology, Faculty of Natural Sciences, Universidad del Rosario, Bogotá, Colombia; University of los Andes, COLOMBIA

## Abstract

Worldwide women have increased their participation in STEM, but we are still far from reaching gender parity. Although progress can be seen at the bachelor’s and master’s level, career advancement of women in research still faces substantial challenges leading to a ‘*leaky pipeline*’ phenomenon (i.e., the continuous decrease of women’s participation at advanced career stages). Latin America exhibits encouraging rates of women participation in research, but the panorama varies across countries and stages in the academic ladder. This study focuses on women’s participation in research in natural sciences in Colombia and investigates career progression, leadership roles, and funding rates by analyzing data on scholarships, grants, rankings, and academic positions. Overall, we found persistent gender imbalances throughout the research ecosystem that were significant using classical statistical analyses. First, although women constitute >50% graduates from bachelors in natural sciences, <40% of researchers in this field are female. Second, women win <30% of research grants, and in turn, their scientific productivity is 2X lower than that of men. Third, because of the less research funding and output women have, their promotion to senior positions in academic and research rankings is slower. In consequence, only ~25% of senior researchers and full professors are women. Fourth, the proportion of women leading research groups and mentoring young scientist in Colombia is <30%. Our study deepens our understanding of gender gaps in STEM research in Colombia, and provides information to design initiatives that effectively target gender disparities by focusing on key areas of intervention, and then gradually building up, rather than tackling structural inequities all at once.

## Introduction

Women have gained more participation in research in recent decades especially in STEM areas, where the number of female graduates is higher than ever before even reaching female-male parity at the bachelor’s and master’s level [1–3]. However, career progression of women in research still faces many challenges, leading to them leaving academia far more frequently than their male counterparts [3]. This disappearance of women as they advance in the scientific career is a global trend that is usually referred to as the ‘*leaky pipeline*’, which is visible as a scissor-shaped curve (i.e., ‘*scissors effect*’) [4–7]. For instance, in 2019 women accounted for 44% of PhDs worldwide, but they only represented 29.3% of active researchers showing a worrying abandonment rate of research professions [8]https://www.zotero.org/google-docs/?sryBxZ. However, in-depth analyses by country or individual discipline show that gender disparities are not the same across all sciences or geographic region. In France and Germany, for example, only ~25% of researchers in STEM are female while in Japan this figure drops as low as 15% [9] Also, a discipline-discerning analysis conducted in Brazil showed that while advancement of women researchers in the biological and health sciences perfectly fits a scissors curve, such pattern is absent in areas such as chemistry, geology, engineering, mathematics, physics, and astronomy where women are consistently underrepresented across all academic stages [10].

Also, women that stay in research careers have a lower research footprint compared to men [11, 12]. Many studies have now shown that there is a bias in the first and last author contributions to high impact journals, and that even when publishing in those journals, the work of women is less cited than that of men [13]. Women also have lower participation in international collaboration networks leading to them having less co-authors and less publication output than men do [2]. Furthermore, there is empirical evidence showing that the probability of women getting research funding is lower not because of the quality of their proposals, but because they are evaluated less favorably as principal investigators [14]. All of these factors contribute to maintaining a glass ceiling for women in science [14, 15], making it difficult for them to reach senior positions which reflects in them being a smaller proportion of faculty and especially tenured faculty [16]. Addressing gender gap issues in research requires continuous and up to date data-driven analyses that help governments, funders, and institutions to implement equity initiatives based on the rigorous identification of the barriers a gender minority faces, and thus allocate resources and support that promote the same outcomes as the non-minoritized counterparts. This is more effective than tackling structural inequities all at once with an equality approach that merely provides the same resources to groups with different characteristics without recognizing the specific challenges each faces [17].

While global studies are important to achieve this goal, regional and local studies are more likely to lead to individual institutional action, yet statistics on women in science at the national level and their use in policy making is still very limited especially in Latin America [18, 19]. In 2016 women accounted for 45.1% of researchers in Latin America and the Caribbean [8], and academies of sciences in the region were among the most gender diverse with most having more than 20% of female members [3]. These statistics show Latin America and the Caribbean as one of the regions with best performance on gender parity worldwide, which is highly encouraging for female researchers. But this is far from homogeneous in the region, with female researchers encompassing from 61% to 30% of researchers in different countries [8]. Nonetheless, in-depth local analyses on female career progression in research are needed to investigate whether such a higher share of female researchers is an honest indicator of improved conditions that allow women to advance in their careers at the same pace as men. In fact, a recent study in Brazil shows that despite an apparent gender equality among researchers, women representation at higher levels of the academic ladder decreases (*scissors effect*) and there is a pervasive gender bias in funding and women representation in research [19]. The most recent gender report of Elsevier was the first to explore global and regional patterns of research participation and career progression for women in science, and included the top three Latin American countries in research output, namely Brazil, Mexico and Argentina [1, 2]. Findings were encouraging. For example, Argentina and Brazil were the countries closest to gender parity in research, with Argentina having the least difference between men and women in research output in 2014–2018 worldwide. Also, Mexico had the largest increase in the proportion of women among inventors (although they remain a minority). The report, however, excluded countries with similar scientific productivity to Argentina, such as Chile and Colombia.

In Colombia, 90% of girls between the ages of 5 and 15 are in school and represent ~50% of secondary school students [20]. Interestingly, women constitute 54% of college students, and bachelor graduates are 56% women and 44% men [21]. Graduates from fields like health and welfare, social sciences, journalism, business administration, law, arts, and humanities are mostly women, accounting for 60–70% of graduates in their field [3]. In contrast, in STEM disciplines the proportion of women graduates is much lower, ranging from 23%-44% [3], except for the natural sciences where women are 54.2% of the bachelor graduates [3]. This female majority persists in early career stages. For example, in 2019 women accounted for 54% of awardees of the national program ‘*Young Researchers*’, which promotes the initiation of a scientific career in Colombia in people younger than 28 [22]. This suggests that natural sciences in Colombia have reached gender parity, at least at the base of the scientific ladder. Nonetheless, women only account for 34.3% of active researchers in natural sciences [3], showing that gender parity does not apply to senior levels. Here, we present data on women’s participation in research in Colombia across career stages investigating both researchers and research groups rankings and funding rates with special focus on the natural sciences. We focused our analyses on self-identified women but could not address other aspects of identity such as race, ethnicity, socio-economic status, sexuality, or disability because this information is not available for the target population. At present, the data available at the national and organizational level only allow us to describe the composition of students, academics and researchers by gender preventing us from characterizing the situation of people in intersections of identity, especially those in minoritized groups. A Spanish version of this contribution can be found in the supplementary material (S1 File).

## Methods

### Data collection

We obtained data on scholarships and research grants awarded by the Colombian Ministry of Science since 2012 (upon request, replied on June 23, 2022). For grants related to the natural sciences, we specifically asked for information on the total amount granted and the gender of the PI (S1 Table). We also requested data on doctoral scholarships awarded for studies in Colombia and abroad separating awardees by gender, and we completed this dataset with information publicly available at https://minciencias.gov.co/la-ciencia-en-cifras/ for the natural sciences. For postdoctoral fellowships in the natural sciences, we downloaded publicly available data from the Ministry’s website (https://minciencias.gov.co/la-ciencia-en-cifras/) keeping only information on fellowships conducted in Colombia.

We also independently downloaded public data from the national platform of research groups (GrupLAC) to obtain information on group ranking and the gender of the group leader. In addition, we obtained the ranking assigned to each researcher in the natural sciences and their self-reported gender over the available years (2013, 2014, 2015, 2017, 2019 and 2021, https://minciencias.gov.co/la-ciencia-en-cifras/ and https://minciencias.gov.co/ciudadano/datosabiertos). Rankings for groups range from A1-D with A1 being the highest rank. Rankings for researchers range from junior to associate to senior to emeritus. Rankings for both groups and researchers are assigned mostly based on productivity (including peer-reviewed papers and student advising), and the criteria used by the Ministry of Science can be found at https://minciencias.gov.co/sites/default/files/upload/convocatoria/anexo_1_-_documento_conceptual_2021.pdf.

We also obtained data from https://minciencias.gov.co/la-ciencia-en-cifras/ on scientific output in the natural sciences by gender per year, that is, the number of research papers, books, book chapters and patents self-reported by each researcher for each evaluation period. However, this data is cumulative so a research output from 2013 would still be counted in 2021.

Additionally, we collected data on the gender of faculty with PhD across 86 universities in Colombia from the National Information System on Higher Education—SNIES (https://snies.mineducacion.gov.co/, S2 Table). As this data does not include classification of faculty per rank and gender, we got this information (upon request) from three major universities in Colombia: (i) Universidad del Tolima, (ii) Universidad de los Andes, and (iii) Universidad del Rosario. We also included public information available for a fourth university: Universidad Nacional [23]. We did not select these universities but rather we restricted our analyses to these four institutions as no other university provided the information requested nor have these data publicly available. The information for Universidad del Tolima and Universidad de los Andes was restricted to the year 2023 (S3 Table), while Universidad Nacional had data for 2015–2019 and Universidad del Rosario for 2015–2021 (S4 Table). At Universidad del Rosario the equivalent rank for “Assistant Professor” is called “Principal Professor” but, for comparison purposes, we renamed it to the former. Due to availability constrains, all this information is for faculty from all disciplines and not only from the natural sciences.

### Statistical analyses

All statistical analyses were performed using the R statistical software V4.3.1 [24]. We tested for significant differences in the number of doctoral and postdoctoral fellowships awarded between genders and across years using ANOVA tests within each category (in Colombia or abroad). Comparisons among genders within years were evaluated using binomial tests as not enough data was available to test for the interaction between the two predictor variables.

We then applied *chi*-square independence tests to evaluate if gender is associated with the rank of a researcher in the Colombian science system (junior, associate, full or emeritus), or the rank of the group a researcher leads (A1-Recognized). In both cases we used post-hoc analyses to determine the specific ranks where significant differences existed.

## Results

**Fig 1:**
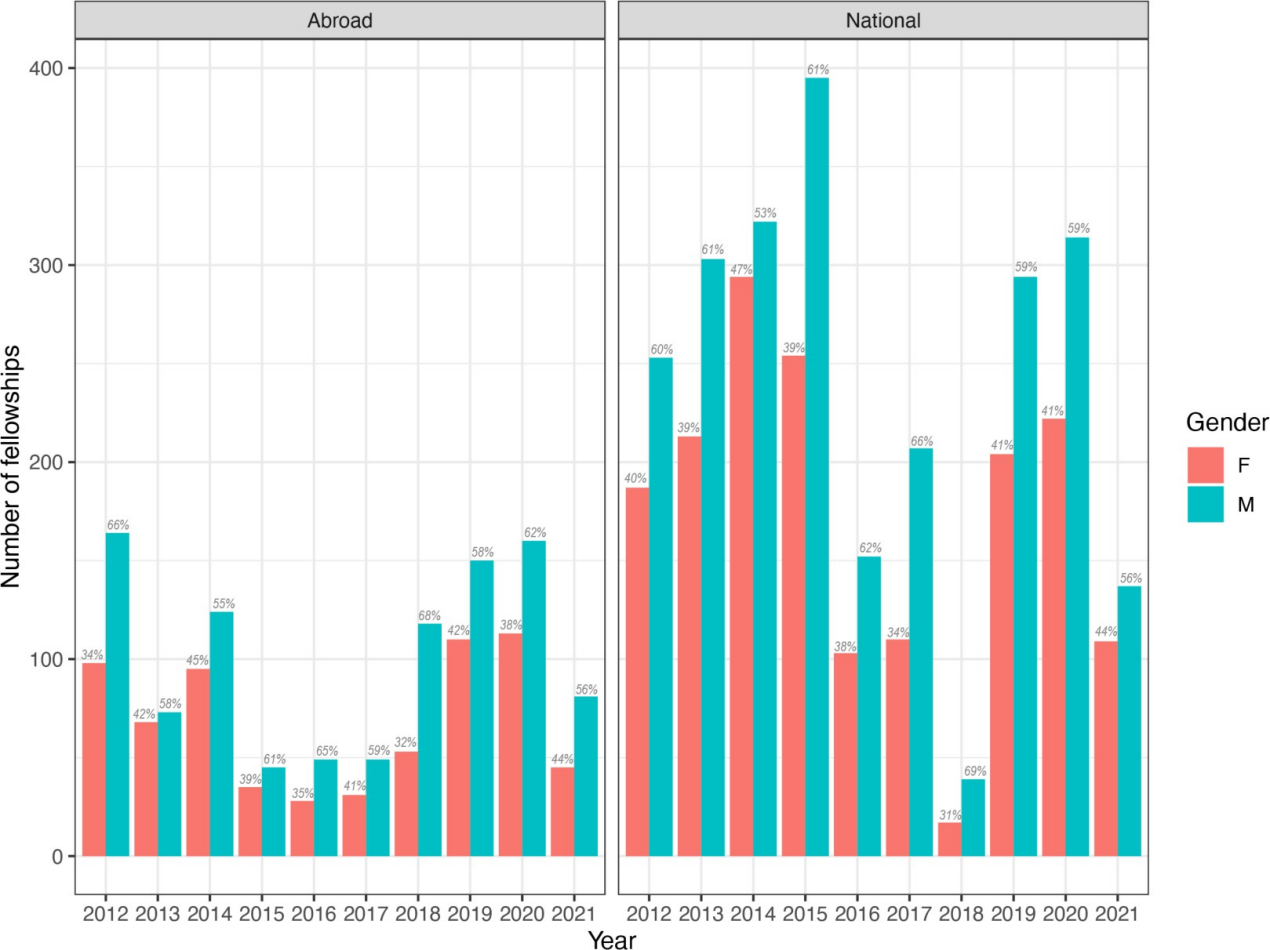
Doctoral scholarships awarded by the Colombian Ministry of Science per year and gender of awardees. Gender is shown as salmon for female awardees and blue for male awardees. Panels represent fellowships for doctoral studies conducted abroad (left) and fellowships for doctoral studies conducted in Colombia (right). The size of the bar indicates the number of scholarships awarded per gender, and for clarity purposes the corresponding percentage is shown on top of each bar.

We found that women are underrepresented in the Colombian research system in the natural sciences, with these differences being present from training to establishment but more pronounced at higher ranks. First, we analyzed data at the doctoral level acknowledging that pursuing a PhD is the most important step for those seeking a career in scientific research in natural sciences. We found that scholarships awarded by the Ministry of Science for PhD programs both in Colombia and abroad have always been given more to male than female candidates (Fig 1). In 2012, for example, 40% of the scholarships for PhDs in Colombia were given to female candidates with this proportion remaining fairly constant over a decade, thus showing little progress over time towards gender parity in scholarship allocation at the national level. Similarly, for PhDs abroad, 34% of scholarships were awarded to women in 2012, and although this proportion has improved in some years, there is no sustained trend towards parity in scholarship allocation over time (Fig 1). Both in PhD programs in Colombia and abroad, we observed the lowest proportion of female candidates receiving funding in 2018 (~31.5%) and a recent increase in 2021 when female awardees reached 44%. There were significant differences in the number of fellowships awarded between genders and years both for PhD scholarships awarded for studies abroad (p = 0.007 for gender and p = 0.003 for years) or in Colombia (p = 0.003 for gender and p = 3x10^-5^ for years). We found that only for 2014 results were not significant with all other years having significantly less awards for women in at least one of the two categories (abroad and in Colombia) and for both categories for five years (2012, 2016, 2018,2019 and 2020, S5 Table).

**Fig 2:**
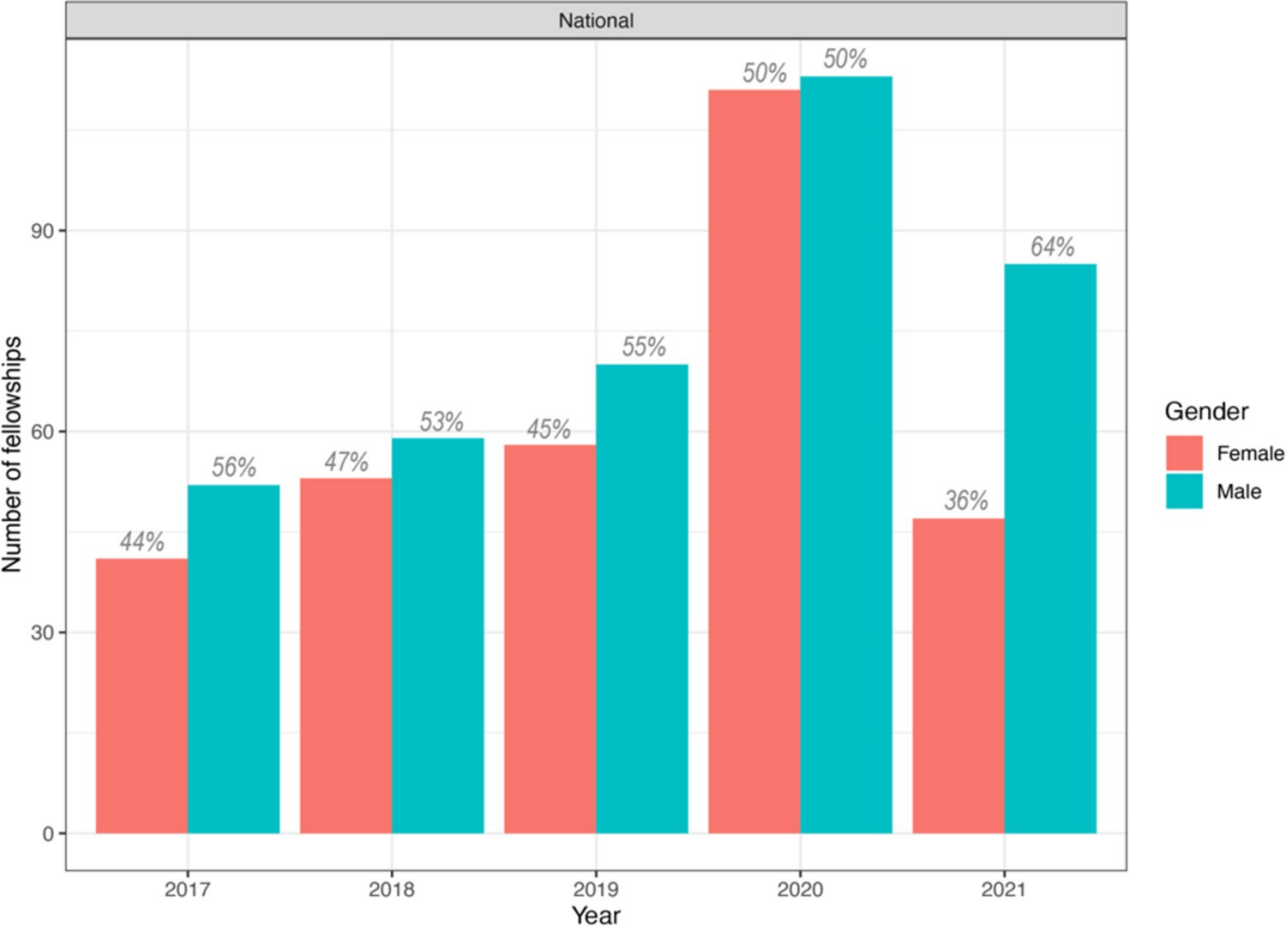
Postdoctoral fellowships awarded by the Ministry of Science per year and gender of awardees. Gender is shown as salmon for female awardees and blue for male awardees. The size of the bar indicates the number of fellowships awarded per gender, and for clarity purposes the corresponding percentage is shown on top of each bar. Data only exists from the year 2017 onwards, as no postdoc funds existed in Colombia before.

Then, we analyzed data at the postdoctoral level which marks the continuation of a scientific career where the researcher deepens the knowledge and skills acquired during the doctoral studies. Interestingly, from the beginning of the postdoctoral funds in Colombia in 2017, there seems to be gender parity in the allocation of fellowships. Thus, although women have received less fellowships across the years, the differences are not significant (p *=* 0.375 for gender, and p = 0.107 for years). However, this has abruptly changed in the most recent call in 2021, where the gap was the highest with 36% of awardees being female and 64% male (Fig 2).

**Fig 3:**
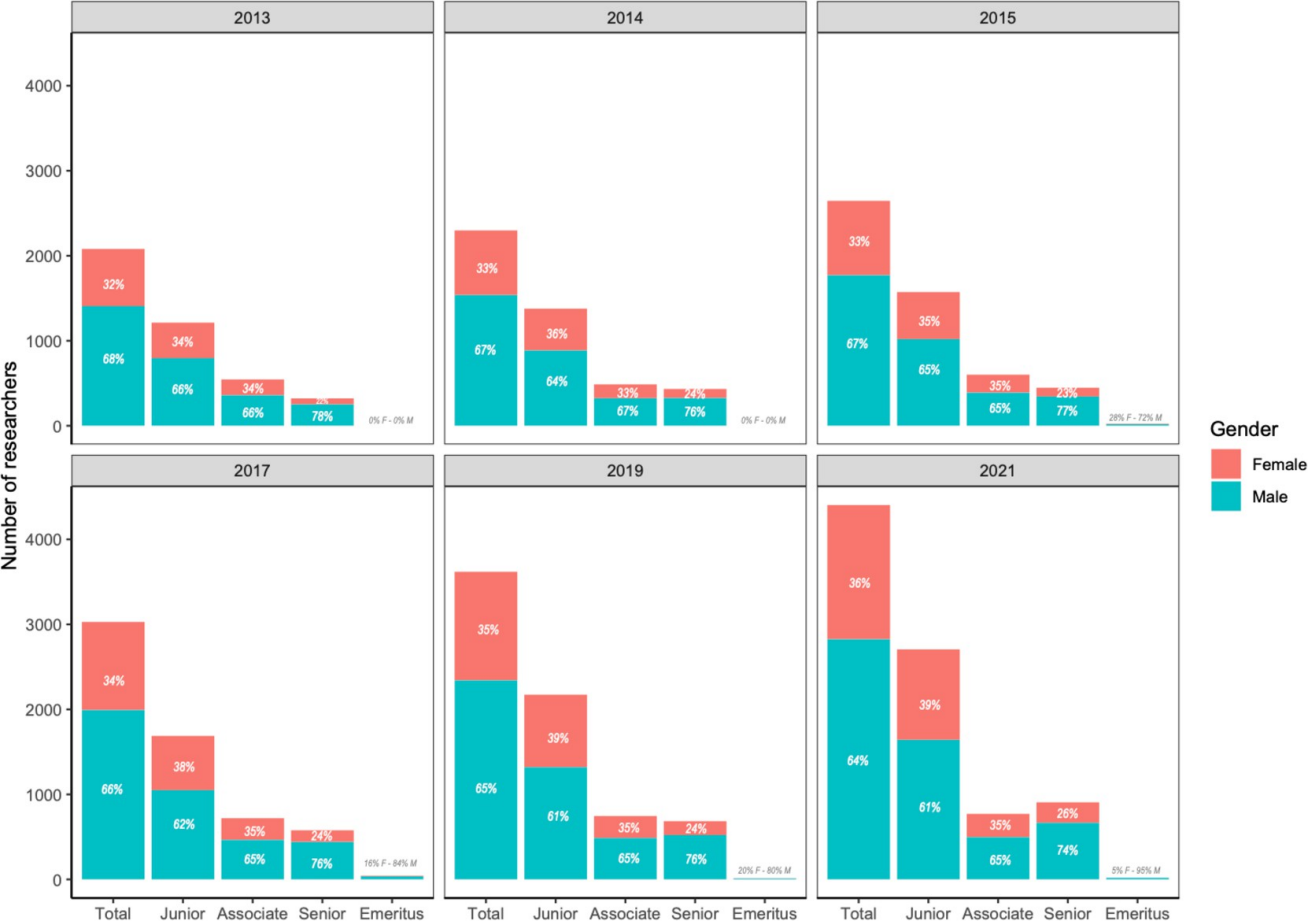
Recognized researchers and their rankings in the Colombian science system between 2013–2021. Researcher gender is self-reported in the government database and is shown as female in salmon and male in blue. Ranks from lowest to highest are: “Investigador Junior”, “Investigador Asociado”, “Investigador Senior” and “Investigador Emérito” shown in the figure with English translations: Junior, Associate, Senior and Emeritus. The bar “Total” shows the combined number of researchers in all ranks. The size of the bar indicates the number of researchers per rank, and for clarity purposes the corresponding percentage is shown within each bar.

In agreement with the above figures, when we looked at the Colombian ranking of researchers in 2021 in all disciplines which included 20,891 researchers with public profile (out of 21,094 registered) and with 20,295 providing their gender (~97%), we found that only 7,837 or ~39% were female (S1 Fig). In consequence, we observed gender and researcher rank were not independent (χ^2^ = 209.35, df = 3, p <2.2x10^-16^), and a post hoc analysis shows significant differences in all but the associate researcher ranks. The representation of women researchers was smaller as the researcher rank increased, going from 42% at the Junior rank to 29% at the Senior rank and 24% at the emeritus rank (S1 Fig). Within the natural sciences, we found an overall growth in the number of recognized female researchers with 2,080 in 2013 and 4,404 in 2021, with the proportion slightly increasing from 32% to 36% in those eight years (Fig 3). For researchers in the natural sciences, gender and researcher rank were not independent for any of the studied years and a post hoc analysis shows significant differences for all ranks across years except for the associate rank where differences were not significant for the years 2017 and 2019 (Fig 3, S6 Table). Within each year, the representation of female researchers was smaller as the researcher rank increased. In 2013 female researchers represented 34% of the researchers at the junior rank, 34% at the associate rank, and 22% at the senior rank (Fig 3). The overall improvement has been modest in almost a decade, and in the last evaluated year, 2021, women’s share of the researcher’s force went from 39% at the junior rank to 35% at the associate rank to 26% at the senior rank and only to 5% at the emeritus rank (Fig 3). Thus, over the last eight years there has been a slight increase in the representation of female researchers in the natural sciences in the different ranks similar to their overall increase, but these results are still far from parity and the increase is strikingly slow.

**Fig 4:**
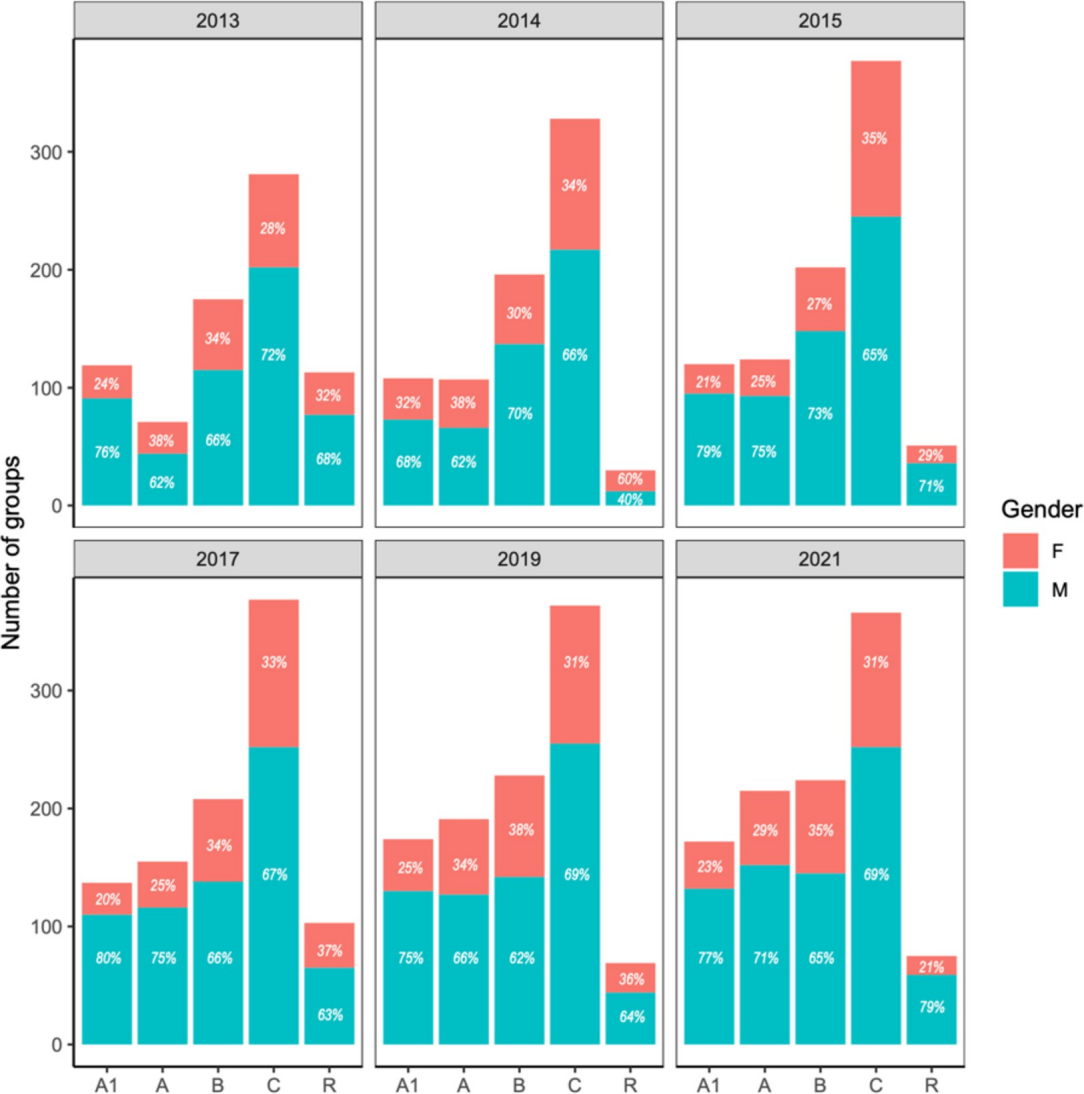
Research groups per rank in the Colombian science system and gender of the group leaders for the years 2013, 2014, 2015, 2017, 2019 and 2021. PI gender was assigned based on the names, with females shown in salmon and male in blue. Ranks from the highest A1 to the lowest C, with “Recognized” (R) just implying recognition of existence without assigning any ranking. It is important to note that not all ranks have been available every year (e.g., A1 was not an option in 2006). The size of the bar indicates the number of groups per rank, and for clarity purposes the corresponding percentage is shown within each bar.

Consequently, although over the last two decades the overall number of recognized research groups in natural sciences in Colombia has increased, the proportion of women that lead those groups has remained exactly the same in almost a decade (Fig 4). For most years analyzed, the gender of the PI is not independent of the group’s rank (S7 Table). For example, in 2014 women were leaders of only 34% of the research groups in natural sciences, with their biggest share in groups ranked as A (38%) and C (34%), and only 32% were ranked as top (rank A1). The tendency has worsen with time. In 2021 not only women still led only 30% of research groups in natural sciences, but their ranking decreased with their groups being mostly ranked as B (35%) and C (31%), and only 23% of them are ranked as A1.

**Fig 5:**
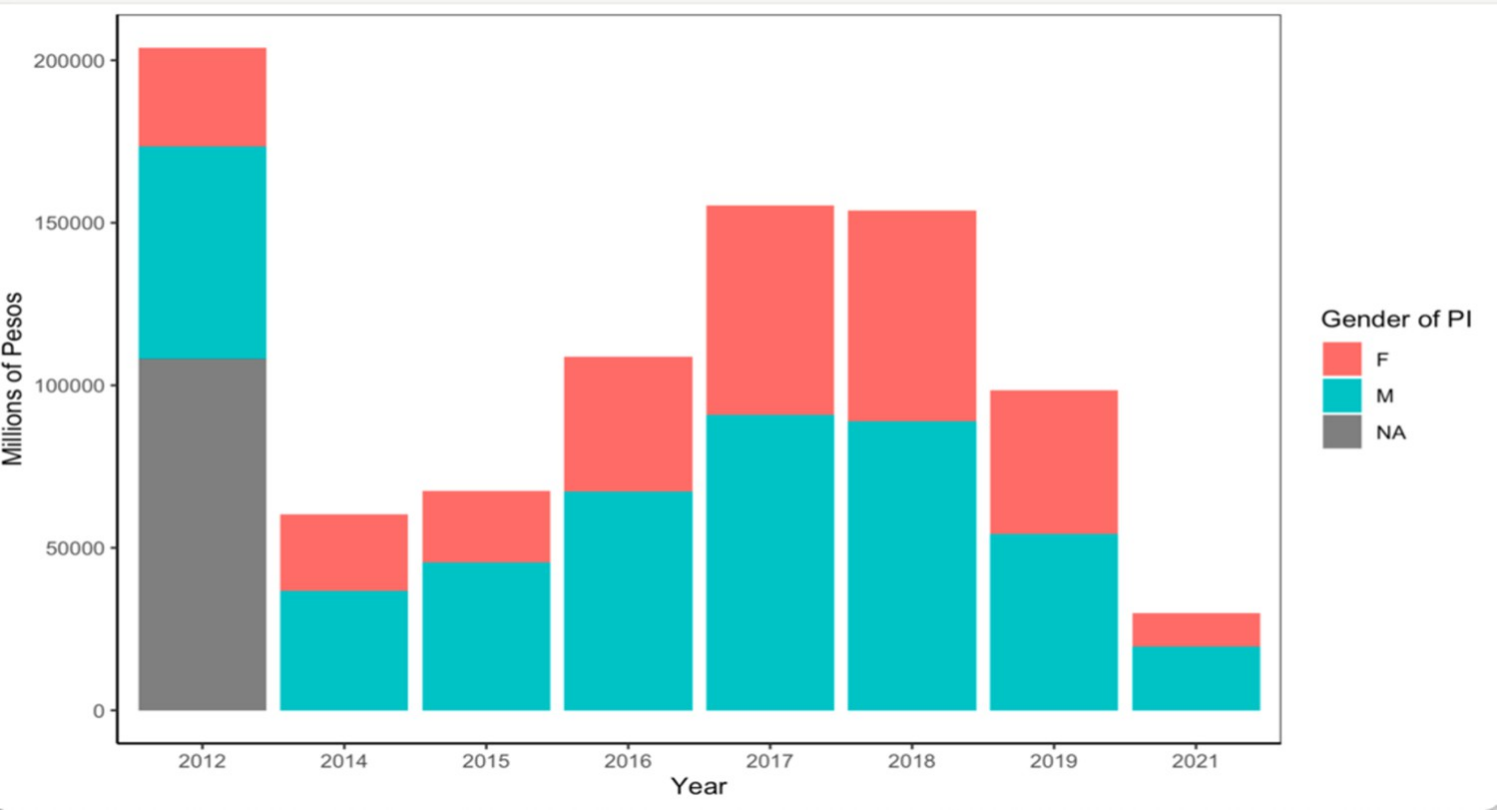
Total amount of funds awarded by the Colombian Ministry of Science and gender of the group leader. Gender information was reported along with project funding information by the Ministry. Amounts awarded are in millions of Colombian pesos (1 USD being equivalent to ~ 4,000 COP as per exchange rate in August 2023). The list of project calls used for the analyses are available in the supplementary material but in short, they correspond to awards in science and not the special “Regalias” programs. For 2012 about half of the proposals did not identify the gender of the group leader.

When we compiled data of calls for research proposals (funding opportunities) in the natural sciences between 2012 and 2021, we found that total amounts awarded each year are variable and seem to be declining. Since 2012 and up to 2021 there is a clear bias with more overall funds awarded to male PIs (Fig 5), and when looking at each year independently, we found that this bias is statistically significant in five out of the eight years analyzed (S8 Table). However, when looking at individual projects, on average, male and female PIs get awarded the same funding per project (S2 Fig) except for 2021, when there is more variation in the individual amounts awarded to male PIs that tend to be lower than those awarded to female group leaders. This agrees with the proportion of scientific output authored by female researchers in Colombia, which is lower than that authored by male researchers. As for research papers, female researchers authored 27% of those reported in 2013 and 29% of those reported in 2021 (S9 Table). However, the data does not allow for distinguishing the position of the author (first and last vs. others) and one paper will be counted as many times as there are coauthors registered. The less amount of research funds female researchers get also agrees with the less mentoring they do. As such, in 2013 women mentored only 22% of PhD theses and 30% of master’s theses, and eight years later this proportion remains unchanged (S10 Table).

Data from the National Information System of Higher Education (SNIES (https://snies.mineducacion.gov.co/) shows that the percentage of women professors for all fields in both public and private universities between 2015 and 2020 represented between 30% and 37% of faculty across all disciplines and ranks, with overall a slightly higher female representation in private than in public universities that was constant across time (S2 Table). There was an increase in female representation between 2015 and 2022 going from 31% to 33% in public universities, and from 34% to 37% in private institutions (S2 Table).

**Fig 6:**
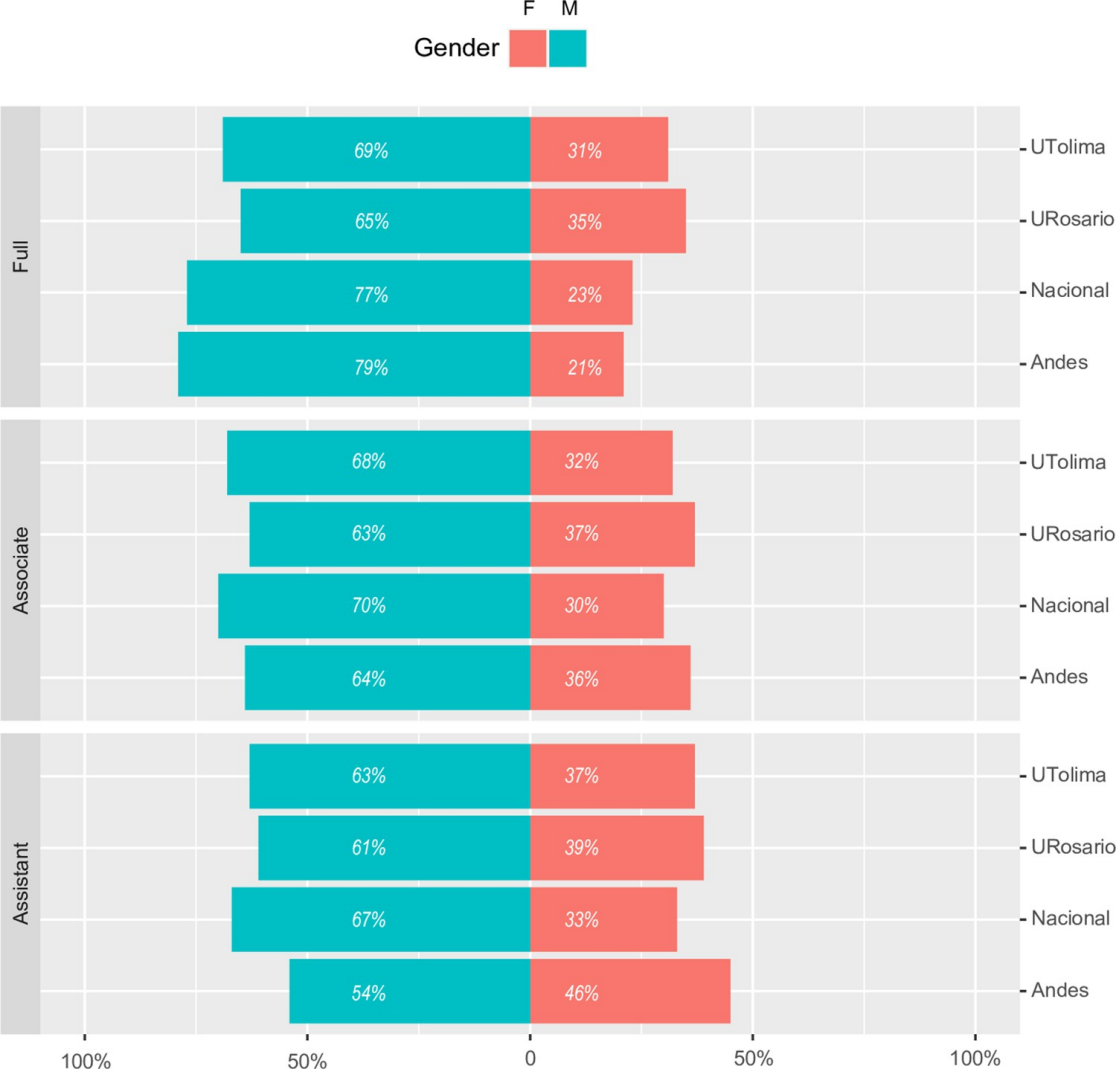
Gender proportion of full-time professors and their rankings. Female in salmon and male in blue. Ranks from lowest to highest are: “Assistant”, “Associate” and “Full”. At Universidad del Rosario the equivalent for “Assistant Professor” is called “Principal Professor” but, for comparison purposes, here we renamed it. Data for Universidad del Tolima (UTolima) and Universidad de los Andes (Andes) is for 2023. Data for Universidad del Rosario (URosario) is for 2021. Data for Universidad Nacional (Nacional) is for 2019 and was not obtained by us but already published in (Bohórquez Montoya et al., 2021). Due to availability constraints, data is for all disciplines and not only for natural sciences. The size of the bar indicates the number of faculty per rank, and for clarity purposes the corresponding percentage is shown within each bar.

The most recent data from individual institutions show a similar picture, with women accounting for only ~30% of academic staff in public universities (i.e., Universidad Nacional and Universidad del Tolima) and ~40% in private universities (i.e., Universidad de los Andes and Universidad del Rosario) (Fig 6; S3 and S4 Tables). In all institutions female professors are underrepresented in all academic ranks, but with a decreased representation in higher academic ranks. For example, ~40% of assistant professors at most universities were women, while at the associate level women professors were ~35% in private universities and ~30% in public ones (Fig 6). In contrast, less than 25% of full professors were women in larger Universities (i.e., Universidad Nacional and Universidad de los Andes), while in the other two institutions this number was barely above 30% (Fig 6). Only at Universidad del Rosario we found more women professors in two ranks, but these are the lowest ranks (“*Auxiliar*”, equivalent to instructors) which are mostly restricted to staff without doctoral degrees, focused on teaching, and with little research time and outputs. We also observed very little progression over time towards more inclusion of women at the higher levels of the academic ladder at least for the two universities with data available for multiple years (S3 and S4 Figs). For example, at Universidad del Rosario the proportion of women professors increased between 2015 and 2016 going from 38% to 45% but has remained mostly constant from there, while at Universidad Nacional there has been no change in the female/male proportion of professors with men being 70% of academic faculty since 2015 (S3 and S4 Figs).

## Discussion

**Fig 7:**
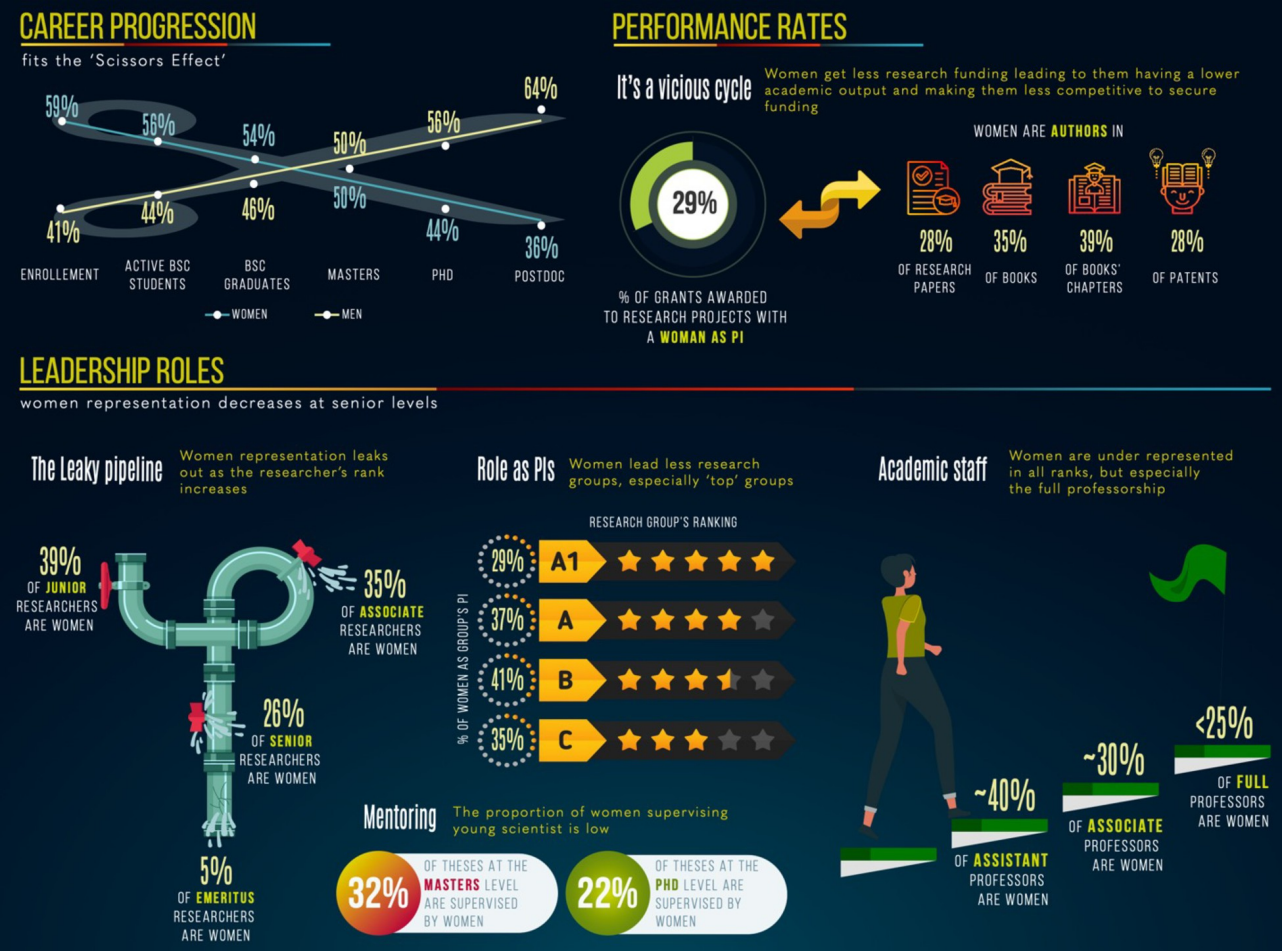
Infographic of women participation in the research ecosystem in Colombia.

Although research in the natural sciences in Colombia have seemingly reached gender parity in early stages of the scientific path (albeit considering only a binary gender classification and no intersectionalities), here we show that women are underrepresented in the research ecosystem, with differences being present from training to establishment but more pronounced at higher ranks (Fig 7). Altogether our findings support that, as in other settings, there is a level beyond which women researchers cannot advance (i.e. a *glass ceiling*).

Despite women being the majority of bachelor graduates in the country [21], we found that at the doctorate level, between 2015–2019, women were awarded only 35% of scholarships in all disciplines [22] and 40% of scholarships in natural sciences (Fig 1) implying a drop in almost 15 percentage points with young female researchers desisting to pursue a career in science. Then, at the postdoctoral level, women constitute on average 44% of awardees of postdoctoral fellowships both across all disciplines and in natural sciences [22]. However, there is not a steady trend towards gender parity, but in contrast, in the most recent call of 2021 only 36% of postdoctoral fellowships in natural sciences were given to women (Fig 2).

While the number of both male and female researchers in Colombia has increased over the last decade [22], we found that women account for less than 40% of researchers in natural sciences in Colombia, and consequently, they only lead 30% of the research groups in the field. When looking at the data in more detail, an even more unequal reality arises. Women are disproportionately represented at the lower levels of the research ladder being mostly ranked as junior researchers that lead groups in the lower ranks of the national ranking system (mostly B and C groups). This is far from being a mere statistical issue and has direct implications in funding opportunities as all national calls give extra points to proposals submitted by research groups in top ranks (A or A1) and senior researchers. Unsurprisingly, Colombian female researchers are less successful in securing grant funding compared to their male peers following global trends [12], which translates into women conducting less research and producing less research outputs. As such, female researchers in natural sciences author ~30% of the publications reported to the Colombian Ministry of Science, which is ~10 percentage points below their overall representation and might be worse as women are less likely to get credit for their work as authorship [12] and men have a tendency to invite more men as coauthors [25] so a single paper may be counted many times for male researchers inflating their participation. This fits the national pattern across all disciplines, where only 28% of research articles have females as authors and most (41%) of the research outputs produced by women are related to outreach [22]. This vicious cycle leads to female researchers in Colombia to have less chances to strengthen their scientific profile and thus they climb the scientific ladder at a much slower pace as men [17, 26]. In consequence, over the course of eight years we only evidenced a slight increase (~4 percentage point) in women’s representation in the national research system for leaders of research groups and recognized researchers.

The gender inequalities observed fit a scissors pattern (Fig 7) and are the result of the expulsion that begins earlier in women’s careers [27]. Causes for this are varied and discussed in depth in the literature, where usually childbearing and other care responsibilities appear as the main explanatory factors [27–29]. While this may be true to an extent, it does not explain how the bias impacts women without children or care responsibilities, and also, it does not explain why the gender gap sharply accentuates when women already hold faculty positions and are not abandoning their scientific career. In fact, the advancement of women researchers in Colombia towards higher academic and scientific ranks is a leaky pipeline where women pour out at specific junctures (Fig 7), a pattern also observed in North America and Europe [30] In the United States, for example, women become principal investigators at a 20% lower rate than men mainly due to them having less publications and citations per publication, which account for ~60% of the gender gap in grant awarding [31]. As in those regions, in Colombia the advancement of researchers to higher ranks heavily relies on research output, grant awarding and scientific mentoring, and women making slower progress than men is likely because of their lower performance in these indicators (Fig 7). This suggests they face gender barriers that limit their careers in a country where conducting research is a challenge itself: government investment in R&D is too low (<0.25% of GDP in the last decade [3] and the armed conflict the country has faced for over 50 years has deepened these barriers, especially affecting women [32].

A possible cause for this is gender bias, *i*.*e*. a prejudice either explicit or implicit based on gender, affecting multiple instances of women’s performance [30]. Gender biases in publication have been confirmed using both experimental and observational research including randomizing names on CVs and abstracts sent for evaluation and evaluating citation numbers and name recognition [33, 34]. Also, the time women have available for research is influenced by gender biases. While it is usually women have less time for research due to their home load, a large proportion of administrative and teaching load in academic settings is assigned to women [35], directly impacting their productivity, job satisfaction and stress levels [36–38]. An additional gender bias comes from junior women researchers having fewer female role models that can act as mentors and support them in understanding the publication and grant application processes as well as introducing them to a network of collaborators [31].

Our data shows that despite the overall increase in participation of women in research, and particularly in natural sciences in Colombia, merely increasing the number of female researchers is not enough to ensure parity, which is a consistent observation across different countries and disciplines [29]. In Colombia, local policies seeking to improve women participation in science have followed the usual approach of motivating the enrollment of women in scientific programs, and at least at the undergraduate and young researcher levels, it has worked [22]. However, the country needs to design and implement new and more assertive policies with special emphasis on: (i) identifying and addressing the drop-out causes for young female researchers, (ii) promote the enrollment of early career researchers with PhD in the scientific ecosystem, and (iii) mitigate the low retention rates and advancement of female researchers in the scientific and academic system. Studies have shown that focusing on young scientists alone is not sufficient to reduce gender imbalance in science [29], and thus, addressing the two latter issues is imperative. Funding calls for postdoctoral fellowships in 2023 from the Ministry of Sciences are, for the first time, giving preference (extra points) to female applicants, as well as minoritized groups, which is expected to motivate the insertion of junior PhD holders in the Colombian research system. The results and impact of these new measures is yet to be evaluated as funds have not been assigned yet. As for obtaining faculty positions at the entry level, hiring calls for full time positions in both in public and private universities, which conduct most of the research in Colombia, usually do not take any measure to encourage female applications making it hard to expect changes towards gender parity in the ranks of researchers and professors. The sole exception is Universidad Industrial de Santander, which in November 2023 launched the first hiring call with gender focus [39] making it the first institution in Colombia to implement specific measures to promote gender equity among their academic staff. Just one of the four universities studied here tends towards gender parity at the entry-level rank for full-time professors (Universidad de los Andes, Fig 6), and only at this institution female/male parity might be expected in higher ranks in the mid and long term (>5 years). However, this hardly is expected in the other institutions. The foreseen persistence of these inequalities at higher ranks are of special concern because highly ranked researchers serve as the main role model for new generations of scientist, and the underrepresentation of women at those positions perpetuate the stereotype that only men can be successful researchers. In fact, a recent study showed that bachelor students in Colombia perceive their male professors as ‘cracks’ while their female professors were perceived as ‘nice’ [40], stressing out the importance that role models play and how crucial is to make it visible the work and accomplishments of women researchers.

Here we show that efforts are falling short in trying to reach gender parity in the Colombian science system and should not focus solely on increasing the number of female researchers, but should address the enrollment, promotion, and advancement women both in the research and academic system, which requires systemic and structural change from governments, funding bodies and academic institutions in order to remove conscious and unconscious biases experienced by women. We suggest some initiatives that are feasible to implement within the Colombian context (Table 1) and stress out the importance of monitoring of their impacts in the mid and long term. Putting these initiatives into action, however, mostly depends on institutions and it is therefore crucial to understand what would motivate governments, funders, and universities to address gender gaps in STEM fields.

**Table 1:** Some suggestions to mitigate gender bias in the research community in Colombia. Here, we present some suggestions from the literature of actions that might help mitigate some of the observed bias in the research community. This is not an extensive list nor a set of silver bullet actions.

Justification	Action	References
Women at the assistant professor level face the pressure of tenure or promotion process under increased family commitments and with no formal mentoring structures.	Create targeted mentoring spaces within scientific societies or universities focused on providing information training on how to structure a promotion application, which increases confidence in the promotion process and reduces the impact of limited access to other networks.	[47]
Women do more internal academic service, which is less valued in promotion processes.	Develop clear administrative expectations by creating service plans for all faculty members (male and female), so it will be easier to detect asymmetric assignments.	[47]
Workshops on “Gender bias habit-breaking intervention” at focal Universities have shown an increase in the probability of making a job offer to a woman.	Promoting the participation of faculty members in “Gender bias habit-breaking" interventions aimed to create awareness, analyze consequences, and propose strategies on gender bias in academic and research environments.	[48, 49]
Current funding allocation mechanisms rely on past achievements as a reliable indicator to judge potential of the applicants, making it hard for young researchers entering the system to compete for funding that allows them to ’launch’ their research program, especially in the absence of startup funds (usually the case in Colombia).	Create a ‘Universal Basic Research Grant’ for assistant professors recently hired that provides a basic allocation of funding each year	[17]
Universities have no real incentives for promoting gender parity	Higher education accreditation should score the commitment of an institution towards reaching gender parity in academic staff.	[17]
Women have more care responsibilities (maternity, caring for elders etc.)	Considering care responsibilities in promotions, rankings and grant evaluations and provide infrastructure for childcare	[50, 51]

From fellowships’ awardees to full-time positions to academic ranks women represent about one third of the total. We acknowledge that biases are present beyond gender and include biases affecting researchers from global south [41], non-native English speakers [42–44], other minoritized groups such as black, indigenous and LGBTIQ+ researchers and different intersectionalities among others [45]. However, here, we chose to focus on gender as this is a category self-reported by Colombian researchers. At present, there is no formal information on other problems that women encounter in academia, namely harassment, mental health issues, racial discrimination, among others, which prevent us from analyzing how these also affect their progress in the academic ladder. Therefore, data must be collected on other minoritized populations including race, socioeconomics and gender identity to identify and act against the biases [46] and we commend the Ministry for having started an effort to collect this data (S11 Table) and include diversity considerations into their more recent calls for training grants.

## Supporting information

S1 Table Grants included in the request for information to the Colombian Ministry of Science, Technology, and Innovation. They were requested by name, call number and year. Request was sent on June 23, 2022.
(DOCX)

S2 Table Gender of faculty with PhD across 86 universities in Colombia (divided by type: Private or public) between 2015 and 2020. Data the National Information System on Higher Education—SNIES (https://snies.mineducacion.gov.co/).
(DOCX)

S3 Table Percentage of female faculty at different ranks for one private university (Universidad de Los Andes) and one public university (Universidad del Tolima) in 2023. Data obtained upon request.
(DOCX)

S4 Table Percentage of female faculty at different ranks for one private university (Universidad del Rosario) from 2015 to 2020. Data obtained upon request. Ranks correspond to the categories used and that institution, and are organized lower to higher as “auxiliar”, “Asistente”, “Principal”,”Asociado”,”Titular”.
(DOCX)

S5 Table Binomial tests for total doctoral fellowships awarded to women for studies abroad or in Colombia. Significant years (with p-value<0.05) are marked with a black asterisk (*) if significant for abroad, a grey asterisk (*) if significant for Colombia and years are in bold if significant for both.
(DOCX)

S6 Table χ^2^ test results for the independence between the rank of researchers in the natural sciences and gender. Significant years (with p-value<0.05) are marked with a *.
(DOCX)

S7 Table χ^2^ test results for the independence between research group rank (A1-D + recognized) and gender of the group leader. Not all group ranks were reported in all years, so degrees of freedom of the test vary among them. Significant years (with p-value<0.05) are marked with a *.
(DOCX)

S8 Table Binomial tests for total grants awarded to women during the period 2012–2021. Only number of grants and not amount awarded. Years with significantly less than 50% grants awarded to women (with p-value<0.05) are marked with a *.
(DOCX)

S9 Table Research output of Colombian researchers between 2013 and 2021, separating by gender and type of output. (DOCX)

S10 Table Thesis supervision (mentoring) at the masters and PhD level of Colombian researchers between 2013 and 2021, separating by gender of the supervisor. (DOCX)

S11 Table Information provided by the Colombian Ministry of Science upon request. Includes information on gender of awardees for science projects, grants to young researchers, grants for field expeditions, fellowships for doctoral and postdoctoral studies.
(XLSX)

S1 Fig Number of recognized researchers and their rankings for the latest recognition call in 2021 by the Colombian Ministry of Science. Researcher gender is self-reported in the government database and is shown here as female in salmon and male in blue. Ranks from lowest to highest are: Junior researcher, associate researcher, senior researcher, and emeritus researcher The bar “Total” shows the combined number of researchers in all ranks.
(DOCX)

S2 Fig Funds awarded by the Colombian Ministry of Science per year and individual project and according to the gender of the group leader between 2012–2021. Gender information was reported along with project funding information by the Ministry. The amounts awarded are in millions of Colombian pesos (1 dollar~ 4100 COP, although exchange rate is variable). List of project calls used for the analyses are available in the supplementary material, but in short, they correspond to awards in science and not the special “Regalias” programs (royalties).
(DOCX)

S3 Fig Gender proportion of full-time professors and their rankings at Universidad del Rosario between 2015–2021. Female in salmon and male in blue. For equivalence and clarity purposes the internal classifications were renamed to be equivalent to the international standard. Ranks from lowest to highest are: Auxiliar 1 (originally called “Auxiliar”), Auxiliar 2 (originally called “Asistente”), Assistant (originally called “Principal”), Associate (originally called “Asociado”), and Full (originally called “Titular”). Auxiliar 1 and 2 are equivalent to instructors. Due to availability constraints, data is for all disciplines and not only for natural sciences.
(DOCX)

S4 Fig Gender proportion of full-time professors and their rankings at Universidad Nacional between 2015–2019. Female in salmon and male in blue. Ranks from lowest to highest are: Auxiliar (originally called “Auxiliar”), (originally called “Asistente”), Associate (originally called “Asociado”), and Full (originally called “Titular”). Auxiliar is equivalent to instructors. Data was already published in Bohórquez Montoya et al., 2021, but due to availability constraints, it is for all disciplines and not only for natural sciences.
(DOCX)

S1 File Full manuscript translated to Spanish. (DOCX)
