# Supplementary material for: Female researchers are under-represented in the Colombian science infrastructure
Source: PLoS One. 2024 Mar 6;19(3):e0298964. doi: 10.1371/journal.pone.0298964 (PMC10917253; doi:10.1371/journal.pone.0298964)
Supplement: S1 Table — They were requested by name, call number and year. Request was sent on June 23, 2022. (DOCX) [file pone.0298964.s001.docx]

Table S1. Grants included in the request for information to the Colombian Ministry of Science, Technology, and Innovation , they were requested by name, call number and year. Request was sent on June 23, 2022

| **Year** | **Call number** | **Call name** |
| --- | --- | --- |
| 2012 | 569 | Convocatoria Nacional para la Conformación de un Banco de Proyectos Elegibles de CTeI Año 2012 |
| 2012 | 576 | Convocatoria Nacional para la Conformación de un Banco de Elegibles de Programas de Ciencia Tecnología e Innovación - CTeI - en Biotecnología y Salud |
| 2014 | 657 | Convocatoria para proyectos de ciencia, tecnología e innovación en salud - 2014 |
| 2014 | 659 | Convocatoria para proyectos de investigación en ciencia, tecnología e innovación en bio: biodiversidad y sus servicios ecosistémicos, medio ambiente marino y aguas continentales, biodiversidad y aprovechamiento sostenible - 2014 |
| 2014 | 660 | Convocatoria para proyectos de ciencia, tecnología e innovación en Geociencias - 2014 |
| 2015 | 710 | Convocatoria para proyectos de investigación en Geociencias - 2015 |
| 2015 | 711 | Convocatoria para proyectos de ciencia, tecnología e innovación en Salud - 2015 |
| 2015 | 712 | Convocatoria para proyectos de investigación en Ciencias Básicas - 2015 |
| 2015 | 714 | Convocatoria para proyectos de investigación, desarrollo tecnológico e innovación en ambiente, océanos y biodiversidad - 2015 |
| 2015 | 725 | Convocatoria para proyectos de investigación en temáticas priorizadas en salud - 2015 |
| 2016 | 744 | Convocatoria para proyectos de Ciencia, Tecnología e Innovación en Salud - 2016 |
| 2016 | 745 | Convocatoria para proyectos de Ciencia, Tecnología e Innovación y su contribución a los retos de país -2016 |
| 2016 | 763 | Convocatoria para proyectos en Ciencia, Tecnología e Innovación en Biodiversidad - 2016 |
| 2017 | 776 | Convocatoria Nacional para la Conformación de un banco de proyectos elegibles de Generación de Nuevo Conocimiento - 2017 |
| 2017 | 777 | Convocatoria para proyectos de Ciencia, Tecnología e Innovación en Salud - 2017 |
| 2017 | 790 | Ideas para el cambio - Ciencia y TIC para la paz |
| 2018 | 807 | Convocatoria para Proyectos de Ciencia, Tecnología e Innovación en Salud 2018 |
| 2018 | 808 | Convocatoria para proyectos de Ciencia, Tecnología e Innovación y su contribución a los retos de país- 2018 |
| 2019 | 842 | Convocatoria para presentar programas de investigación en temáticas priorizadas en ciencias médicas y de la salud |
| 2019 | 844 | Convocatoria pacto para la generación de nuevo conocimiento a través de proyectos de investigación científica en ciencias médicas y de la salud |
| 2019 | 866 | Convocatoria Expediciones Científicas Nacionales y Fortalecimiento de Colecciones Biológicas |
| 2020 | 874 | Convocatoria para el fortalecimiento de proyectos en ejecución de CTeI en ciencias de la salud con talento joven e impacto regional |
| 2021 | 896 | Convocatoria fortalecimiento de capacidades regionales de investigación en salud |
| 2021 | 900 | Convocatoria Ideas para el Cambio: Construcción social del conocimiento para la gestión del cambio climático |
| **Formación de capital humano en CTeI** | | |
| 2012 | 568 | Convocatoria Nacional para estudios de Doctorado en el Exterior Año 2012 Conformación de un Banco de Elegibles |
| 2012 | 567 | Convocatoria Nacional para estudios de Doctorado en Colombia año 2012 Conformación de un Banco de Elegibles |
| 2013 | 617 | Convocatoria para conformar bancos de elegibles para formación de alto nivel para la ciencia, la tecnología y la innovación (semilleros y jóvenes investigadores, doctorados nacionales y en el exterior) |
| 2014 | 646 | Doctorados en el exterior 2014 |
| 2014 | 647 | Doctorados nacionales 2014 |
| 2014 | 696 | Convocatoria es Tiempo de Volver 2014 segundo corte |
| 2015 | 727 | Convocatoria Doctorados Nacionales 2015 |
| 2015 | 728 | Convocatoria Doctorados en el Exterior 2015 |
| 2016 | 756 | Convocatoria de Doctorados en el Exterior 2016 |
| 2016 | 757 | Convocatoria Doctorados Nacionales 2016 |
| 2016 | 767 | Convocatoria Doctorados Nacionales Jóvenes- 2016 |
| 2017 | 783 | Convocatoria de Doctorados en el Exterior 2017 |
| 2017 | 784 | Convocatoria programa de Estancias Postdoctorales beneficiarios Colciencias 2017 |
| 2018 | 811 | Programa de estancias postdoctorales para beneficiarios de formación Colciencias en entidades del SNCTeI |
| 2019 | 848 | Convocatoria Programa de Estancias Postdoctorales en entidades del SNCTeI 2019 |
| 2019 | 860 | Convocatoria de doctorados en el exterior |
| 2020 | 885 | Convocatoria Doctorados en el Exterior |
| 2021 | 906 | Convocatoria de Doctorados en el Exterior |
| **Medición de grupos de investigación e investigadores** | | |
| 2012 | 598 | Invitación para el Reconocimiento de Grupos de Investigación en Ciencia, Tecnología e Innovación Año 2012 |
| 2013 | 640 | Convocatoria nacional para el reconocimiento y medición de grupos de investigación, desarrollo tecnológico y/o innovación y para el reconocimiento de investigadores del sistema nacional de ciencia, tecnología e innovación. 2013 |
| 2014 | 693 | Convocatoria Nacional para el Reconocimiento y Medición de Grupos de Investigación, Desarrollo Tecnológico o de Innovación y para el Reconocimiento de Investigadores del SNCTeI – 2014 |
| 2017 | 781 | Convocatoria nacional para el reconocimiento y medición de Grupos de Investigación, Desarrollo Tecnológico o de Innovación y para el reconocimiento de Investigadores del Sistema Nacional de Ciencia, Tecnología e Innovación – SNCTeI 2017 |
| 2018 | 833 | Convocatoria Nacional para el reconocimiento y medición de grupos de investigación, desarrollo tecnológico o de innovación y para el reconocimiento de investigadores del Sistema Nacional de Ciencia, Tecnología e Innovación – SNCTeI, 2018 |
| 2021 | 894 | Convocatoria nacional para el reconocimiento y medición de grupos de investigación, desarrollo tecnológico o de innovación y para el reconocimiento de investigadores del Sistema Nacional de Ciencia, Tecnología e Innovación - SNCTI 2021 |
