# Supplementary material for: Female researchers are under-represented in the Colombian science infrastructure
Source: PLoS One. 2024 Mar 6;19(3):e0298964. doi: 10.1371/journal.pone.0298964 (PMC10917253; doi:10.1371/journal.pone.0298964)
Supplement: S2 Table — Data the National Information System on Higher Education—SNIES (https://snies.mineducacion.gov.co/). (DOCX) [file pone.0298964.s002.docx]

**Table S2.** Gender of faculty with PhD across 86 universities in Colombia (divided by type: private or public) between 2015 and. Data the National Information System on Higher Education - SNIES (<https://snies.mineducacion.gov.co/>

| **Type** | **Year** | **Male** | **Female** | **Percentage of female** |
| --- | --- | --- | --- | --- |
| Private | 2015 | 1804 | 926 | 34% |
| Public | 2015 | 2453 | 1105 | 31% |
| Private | 2016 | 2074 | 1123 | 35% |
| Public | 2016 | 2688 | 1226 | 31% |
| Private | 2017 | 2560 | 1407 | 35% |
| Public | 2017 | 3119 | 1449 | 32% |
| Private | 2018 | 2729 | 1530 | 36% |
| Public | 2018 | 3294 | 1593 | 33% |
| Private | 2019 | 2975 | 1678 | 36% |
| Public | 2019 | 3546 | 1710 | 33% |
| Private | 2020 | 2804 | 1679 | 38% |
| Public | 2020 | 3797 | 1861 | 33% |
