# Supplementary material for: Female researchers are under-represented in the Colombian science infrastructure
Source: PLoS One. 2024 Mar 6;19(3):e0298964. doi: 10.1371/journal.pone.0298964 (PMC10917253; doi:10.1371/journal.pone.0298964)
Supplement: S3 Table — Data obtained upon request. (DOCX) [file pone.0298964.s003.docx]

**Table S3.** Percentage of female faculty at different ranks for one private university (Universidad de Los Andes) and one public university (Universidad del Tolima) in 2023. Data obtained upon request.

| **University** | **Rank** | **Percentage of female** |
| --- | --- | --- |
| Universidad de Los Andes | Assistant | 45.27% |
|  | Associate | 36.15% |
|  | Full | 20.69% |
|  | Overall | 37% |
| Universidad del Tolima | *Auxiliar* | 29% |
|  | Assistant | 37% |
|  | Associate | 32% |
|  | Full | 30.56% |
|  | Overall | 33.33% |
