# Supplementary material for: Female researchers are under-represented in the Colombian science infrastructure
Source: PLoS One. 2024 Mar 6;19(3):e0298964. doi: 10.1371/journal.pone.0298964 (PMC10917253; doi:10.1371/journal.pone.0298964)
Supplement: S4 Table — Data obtained upon request. Ranks correspond to the categories used and that institution, and are organized lower to higher as “auxiliar”, “Asistente”, “Principal”,”Asociado”,”Titular”. (DOCX) [file pone.0298964.s004.docx]

**Table S4.** Percentage of female faculty at different ranks for one private university (Universidad del Rosario) from 2015 to 2020. Data obtained upon request. Ranks correspond to the categories used and that institution, and are organized lower to higher as “auxiliar”, “Asistente”, “Principal”,”Asociado”,”Titular”

| **Year** | **Rank** | **Percentage Female** |
| --- | --- | --- |
| 2015 | Auxiliar | 69.64% |
|  | Asistente | 56.00% |
|  | Principal | 21.88% |
|  | Asociado | 37.21% |
|  | Titular | 21.88% |
| 2016 | Auxiliar | 68.66% |
|  | Asistente | 57.69% |
|  | Principal | 43.93% |
|  | Asociado | 39.13% |
|  | Titular | 23.88% |
| 2017 | Auxiliar | 68.92% |
|  | Asistente | 56.67% |
|  | Principal | 40.16% |
|  | Asociado | 38.00% |
|  | Titular | 23.53% |
| 2018 | Auxiliar | 68.25% |
|  | Asistente | 55.26% |
|  | Principal | 40.00% |
|  | Asociado | 41.18% |
|  | Titular | 32.05% |
| 2019 | Auxiliar | 66.04% |
|  | Asistente | 53.33% |
|  | Principal | 39.84% |
|  | Asociado | 38.71% |
|  | Titular | 32.88% |
| 2020 | Auxiliar | 63.33% |
|  | Asistente | 54.00% |
|  | Principal | 38.97% |
|  | Asociado | 38.10% |
|  | Titular | 34.29% |
| 2021 | Auxiliar | 57.14% |
|  | Asistente | 68.52% |
|  | Principal | 39.23% |
|  | Asociado | 37.04% |
|  | Titular | 34.72% |
