# Supplementary material for: Female researchers are under-represented in the Colombian science infrastructure
Source: PLoS One. 2024 Mar 6;19(3):e0298964. doi: 10.1371/journal.pone.0298964 (PMC10917253; doi:10.1371/journal.pone.0298964)
Supplement: S5 Table — Significant years (with p-value<0.05) are marked with a black asterisk (*) if significant for abroad, a grey asterisk (*) if significant for Colombia and years are in bold if significant for both. (DOCX) [file pone.0298964.s005.docx]

**Table S5.** Binomial tests for total doctoral fellowships awarded to women for studies abroad or in Colombia. Significant years (with p-value<0.05) are marked with a black asterisk (*) if significant for abroad, a grey asterisk (*) if significant for Colombia and years are in bold if significant for both.

|  | **Abroad** | | | **Colombia** | | |
| --- | --- | --- | --- | --- | --- | --- |
| **Year** | **Women** | **Total** | **Binomial test** | **Women** | **Total** | **Binomial test** |
| **2012** | 187 | 440 | 0.002 | 98 | 262 | 5.45*10^-5^ |
| 2013* | 213 | 516 | 8.59 *10^-5^ | 68 | 141 | 0.74 |
| 2014 | 294 | 616 | 0.277 | 95 | 219 | 0.058 |
| 2015* | 254 | 649 | 3.46*10^-8^ | 35 | 80 | 0.314 |
| **2016** | 103 | 255 | 0.003 | 28 | 77 | 0.022 |
| 2017* | 110 | 317 | 5.54*10^-8^ | 31 | 80 | 0.057 |
| **2018** | 17 | 56 | 0.005 | 53 | 171 | 7.35*10^-7^ |
| **2019** | 204 | 498 | 6.38*10^-5^ | 110 | 260 | 0.015 |
| **2020** | 222 | 536 | 8.16*10^-5^ | 113 | 273 | 0.005 |
| 2021* | 109 | 246 | 0.085 | 45 | 126 | 0.002 |
