# Supplementary material for: Female researchers are under-represented in the Colombian science infrastructure
Source: PLoS One. 2024 Mar 6;19(3):e0298964. doi: 10.1371/journal.pone.0298964 (PMC10917253; doi:10.1371/journal.pone.0298964)
Supplement: S6 Table — Significant years (with p-value<0.05) are marked with a *. (DOCX) [file pone.0298964.s006.docx]

**Table S6. χ^2^ test results for the independence between the rank of researchers in the natural sciences and gender.** Significant years (with p-value<0.05) are marked with a *.

| **Year** | **χ^2^** | **Degrees of freedom** | **p-value** |
| --- | --- | --- | --- |
| 2013* | 135 | 3 | < 2.2e-16 |
| 2014* | 144 | 3 | < 2.2e-16 |
| 2015* | 107 | 3 | < 2.2e-16 |
| 2017* | 140 | 3 | < 2.2e-16 |
| 2019* | 192 | 3 | < 2.2e-16 |
| 2021* | 204 | 3 | < 2.2e-16 |
