# Supplementary material for: Female researchers are under-represented in the Colombian science infrastructure
Source: PLoS One. 2024 Mar 6;19(3):e0298964. doi: 10.1371/journal.pone.0298964 (PMC10917253; doi:10.1371/journal.pone.0298964)
Supplement: S7 Table — Not all group ranks were reported in all years, so degrees of freedom of the test vary among them. Significant years (with p-value<0.05) are marked with a *. (DOCX) [file pone.0298964.s007.docx]

**Table S7. χ^2^ test results for the independence between research group rank (A1-C + recognized) and gender of the group leader.** Group ranks were not reported for all. Significant years (with p-value<0.05) are marked with a *.

| **Year** | **χ^2^** | **Degrees of freedom** | **p-value** |
| --- | --- | --- | --- |
| 2013 | 6.67 | 4 | 0.15 |
| 2014* | 11.29 | 4 | 0.024 |
| 2015* | 11.81 | 4 | 0.02 |
| 2017* | 13.82 | 4 | 0.008 |
| 2019 | 7.67 | 4 | 0.1 |
| 2021* | 9.65 | 4 | 0.047 |
