# Supplementary material for: Female researchers are under-represented in the Colombian science infrastructure
Source: PLoS One. 2024 Mar 6;19(3):e0298964. doi: 10.1371/journal.pone.0298964 (PMC10917253; doi:10.1371/journal.pone.0298964)
Supplement: S8 Table — Only number of grants and not amount awarded. Years with significantly less than 50% grants awarded to women (with p-value<0.05) are marked with a *. (DOCX) [file pone.0298964.s008.docx]

**Table S8.** Binomial tests for total grants awarded to women during the period 2012-2021. Only number of grants and not amount awarded. Years with significantly less than 50% grants awarded to women (with p-value<0.05) are marked with a *.

| **Year** | **Women** | **Total** | **Binomial test** |
| --- | --- | --- | --- |
| 2012* | 48 | 131 | 0.002829 |
| 2014 | 54 | 130 | 0.06509 |
| 2015* | 49 | 153 | 1.03 x10^-5^ |
| 2016* | 70 | 184 | 0.001456 |
| 2017* | 91 | 212 | 0.04615 |
| 2018* | 86 | 205 | 0.02518 |
| 2019 | 56 | 127 | 0.214 |
| 2021 | 8 | 27 | 0.05224 |
