# Supplementary material for: Female researchers are under-represented in the Colombian science infrastructure
Source: PLoS One. 2024 Mar 6;19(3):e0298964. doi: 10.1371/journal.pone.0298964 (PMC10917253; doi:10.1371/journal.pone.0298964)
Supplement: S9 Table — (DOCX) [file pone.0298964.s009.docx]

**Table S9.** Research output of Colombian researchers between 2013 and 2021, separating by gender and type of output.

| **Year** | **Gender** | **Type of output** | **Number** |
| --- | --- | --- | --- |
| 2013 | Female | Research paper | 4902 |
| 2013 | Female | Research book | 338 |
| 2013 | Female | Chapters in research books | 85 |
| 2013 | Female | Patent | 9 |
| 2013 | Male | Research paper | 13149 |
| 2013 | Male | Research book | 918 |
| 2013 | Male | Chapters in research books | 290 |
| 2013 | Male | Patent | 29 |
| 2014 | Female | Research paper | 7465 |
| 2014 | Female | Research book | 148 |
| 2014 | Female | Chapters in research books | 301 |
| 2014 | Female | Patent | 19 |
| 2014 | Male | Research paper | 19886 |
| 2014 | Male | Research book | 265 |
| 2014 | Male | Chapters in research books | 755 |
| 2014 | Male | Patent | 27 |
| 2015 | Female | Research paper | 8621 |
| 2015 | Female | Research book | 223 |
| 2015 | Female | Chapters in research books | 294 |
| 2015 | Female | Patent | 41 |
| 2015 | Male | Research paper | 23787 |
| 2015 | Male | Research book | 387 |
| 2015 | Male | Chapters in research books | 709 |
| 2015 | Male | Patent | 101 |
| 2017 | Female | Research paper | 11212 |
| 2017 | Female | Research book | 303 |
| 2017 | Female | Chapters in research books | 431 |
| 2017 | Female | Patent | 36 |
| 2017 | Male | Research paper | 28330 |
| 2017 | Male | Research book | 570 |
| 2017 | Male | Chapters in research books | 753 |
| 2017 | Male | Patent | 56 |
| 2019 | Female | Research paper | 14386 |
| 2019 | Female | Research book | 396 |
| 2019 | Female | Chapters in research books | 722 |
| 2019 | Female | Patent | 82 |
| 2019 | Male | Research paper | 36069 |
| 2019 | Male | Research book | 780 |
| 2019 | Male | Chapters in research books | 1277 |
| 2019 | Male | Patent | 155 |
| 2021 | Female | Research paper | 17204 |
| 2021 | Female | Research book | 510 |
| 2021 | Female | Chapters in research books | 1219 |
| 2021 | Female | Patent | 108 |
| 2021 | Male | Research paper | 43172 |
| 2021 | Male | Research book | 928 |
| 2021 | Male | Chapters in research books | 1871 |
| 2021 | Male | Patent | 267 |
