# Supplementary material for: Female researchers are under-represented in the Colombian science infrastructure
Source: PLoS One. 2024 Mar 6;19(3):e0298964. doi: 10.1371/journal.pone.0298964 (PMC10917253; doi:10.1371/journal.pone.0298964)
Supplement: S10 Table — (DOCX) [file pone.0298964.s010.docx]

**Table S10.** Thesis supervision (mentoring) at the masters and PhD level of Colombian researchers between 2013 and 2021, separating by gender of the supervisor.

| **Year** | **Gender** | **Type of mentoring** | **Number** | **Proportion** |
| --- | --- | --- | --- | --- |
| 2013 | Female | PhD theses | 184 | 22% |
| 2013 | Male | PhD theses | 639 | 78% |
| 2013 | Female | MSc theses | 1639 | 30% |
| 2013 | Male | MSc theses | 3797 | 70% |
| 2014 | Female | PhD theses | 179 | 21% |
| 2014 | Male | PhD theses | 658 | 79% |
| 2014 | Female | MSc theses | 1630 | 28% |
| 2014 | Male | MSc theses | 4266 | 72% |
| 2015 | Female | PhD theses | 239 | 21% |
| 2015 | Male | PhD theses | 924 | 79% |
| 2015 | Female | MSc theses | 1895 | 27% |
| 2015 | Male | MSc theses | 5069 | 73% |
| 2017 | Female | PhD theses | 266 | 24% |
| 2017 | Male | PhD theses | 861 | 76% |
| 2017 | Female | MSc theses | 2462 | 29% |
| 2017 | Male | MSc theses | 5924 | 71% |
| 2019 | Female | PhD theses | 289 | 23% |
| 2019 | Male | PhD theses | 968 | 77% |
| 2019 | Female | MSc theses | 2552 | 29% |
| 2019 | Male | MSc theses | 6178 | 71% |
| 2021 | Female | PhD theses | 475 | 22% |
| 2021 | Male | PhD theses | 1693 | 78% |
| 2021 | Female | MSc theses | 4339 | 32% |
| 2021 | Male | MSc theses | 9189 | 68% |
