# Supplementary material for: Female researchers are under-represented in the Colombian science infrastructure
Source: PLoS One. 2024 Mar 6;19(3):e0298964. doi: 10.1371/journal.pone.0298964 (PMC10917253; doi:10.1371/journal.pone.0298964)
Supplement: S1 Fig — Researcher gender is self-reported in the government database and is shown here as female in salmon and male in blue. Ranks from lowest to highest are: Junior researcher, associate researcher, senior researcher, and emeritus researcher The bar “Total” shows the combined number of researchers in all ranks. (DOCX) [file pone.0298964.s012.docx]

**Figure S1. Number of recognized researchers and their rankings for the latest recognition call in 2021 by the Colombian Ministry of Science.** Researcher gender is self-reported in the government database and is shown here as female in salmon and male in blue. Ranks from lowest to highest are: Junior researcher, associate researcher, senior researcher, and emeritus researcher The bar “Total” shows the combined number of researchers in all ranks.
