# Supplementary material for: Female researchers are under-represented in the Colombian science infrastructure
Source: PLoS One. 2024 Mar 6;19(3):e0298964. doi: 10.1371/journal.pone.0298964 (PMC10917253; doi:10.1371/journal.pone.0298964)
Supplement: S2 Fig — Gender information was reported along with project funding information by the Ministry. The amounts awarded are in millions of Colombian pesos (1 dollar~ 4100 COP, although exchange rate is variable). List of project calls used for the analyses are available in the supplementary material, but in short, they correspond to awards in science and not the special “Regalias” programs (royalties). (DOCX) [file pone.0298964.s013.docx]

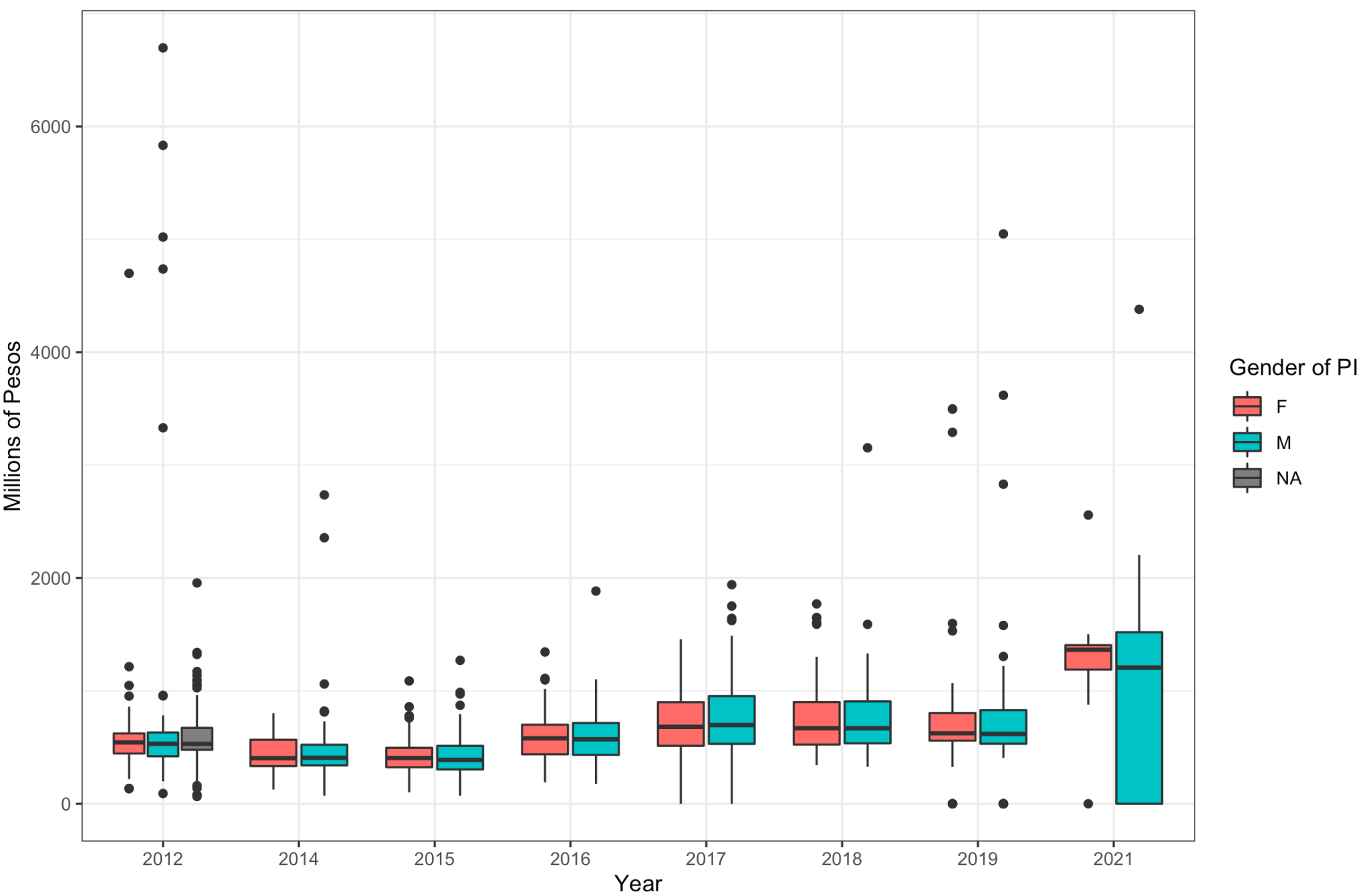


**Figure S2.** **Funds awarded by the Colombian Ministry of Science per year and individual project and according to the gender of the group leader between 2012-2021**. Gender information was reported along with project funding information by the Ministry. The amounts awarded are in millions of Colombian pesos (1 dollar~ 4100 COP, although exchange rate is variable). List of project calls used for the analyses are available in the supplementary material, but in short, they correspond to awards in science and not the special “Regalias” programs (royalties).
