# Supplementary material for: Female researchers are under-represented in the Colombian science infrastructure
Source: PLoS One. 2024 Mar 6;19(3):e0298964. doi: 10.1371/journal.pone.0298964 (PMC10917253; doi:10.1371/journal.pone.0298964)
Supplement: S3 Fig — Female in salmon and male in blue. For equivalence and clarity purposes the internal classifications were renamed to be equivalent to the international standard. Ranks from lowest to highest are: Auxiliar 1 (originally called “Auxiliar”), Auxiliar 2 (originally called “Asistente”), Assistant (originally called “Principal”), Associate (originally called “Asociado”), and Full (originally called “Titular”). Auxiliar 1 and 2 are equivalent to instructors. Due to availability constraints, data is for all disciplines and not only for natural sciences. (DOCX) [file pone.0298964.s014.docx]

**Figure S3. Gender proportion of full-time professors and their rankings at Universidad del Rosario between 2015-2021.** Female in salmon and male in blue. For equivalence and clarity purposes the internal classifications were renamed to be equivalent to the international standard. Ranks from lowest to highest are: Auxiliar 1 (originally called “Auxiliar”), Auxiliar 2 (originally called “Asistente”), Assistant (originally called “Principal”), Associate (originally called “Asociado”), and Full (originally called “Titular”). Auxiliar 1 and 2 are equivalent to instructors. Due to availability constraints, data is for all disciplines and not only for natural sciences.
