# Supplementary material for: Female researchers are under-represented in the Colombian science infrastructure
Source: PLoS One. 2024 Mar 6;19(3):e0298964. doi: 10.1371/journal.pone.0298964 (PMC10917253; doi:10.1371/journal.pone.0298964)
Supplement: S4 Fig — Female in salmon and male in blue. Ranks from lowest to highest are: Auxiliar (originally called “Auxiliar”), (originally called “Asistente”), Associate (originally called “Asociado”), and Full (originally called “Titular”). Auxiliar is equivalent to instructors. Data was already published in Bohórquez Montoya et al., 2021, but due to availability constraints, it is for all disciplines and not only for natural sciences. (DOCX) [file pone.0298964.s015.docx]

**Figure S4. Gender proportion of full-time professors and their rankings at Universidad Nacional between 2015-2019.** Female in salmon and male in blue. Ranks from lowest to highest are: Auxiliar (originally called “Auxiliar”), (originally called “Asistente”), Associate (originally called “Asociado”), and Full (originally called “Titular”). Auxiliar is equivalent to instructors. Data was already published in Bohórquez Montoya et al., 2021, but due to availability constraints, it is for all disciplines and not only for natural sciences.
