# Supplementary material for: Female researchers are under-represented in the Colombian science infrastructure
Source: PLoS One. 2024 Mar 6;19(3):e0298964. doi: 10.1371/journal.pone.0298964 (PMC10917253; doi:10.1371/journal.pone.0298964)
Supplement: S1 File — (DOCX) [file pone.0298964.s016.docx]

**Las mujeres investigadoras están subrepresentadas en la infraestructura de investigación colombiana**

Andrea Paz^1,2^, Carolina Pardo-Díaz^3^

^1^ Department of Environmental Systems Science, Institute of Integrative Biology, ETH Zürich, Zürich, Switzerland.

^2^ Département de Sciences Biologiques, Université de Montréal, Montréal, Canada

^3^ Departamento de Biología, Facultad de Ciencias Naturales, Universidad del Rosario, Bogotá, Colombia.

**Resumen**

A nivel mundial las mujeres han aumentado su participación en STEM, pero aún estamos lejos de alcanzar la paridad de género. Aunque se observa progreso a nivel de pregrado y maestría, el avance en la carrera investigativa de las mujeres aún enfrenta grandes desafíos que generan a un fenómeno de "*tubería con fugas*" (es decir, la disminución continua de la participación de las mujeres en etapas avanzadas de la carrera). América Latina muestra tasas alentadoras de participación de mujeres en investigación, pero el panorama varía entre países y etapas de la escalera académica. Este estudio se centra en la participación de las mujeres en investigación en ciencias naturales en Colombia e investiga su avance profesional, roles de liderazgo y tasas de financiación a través del análisis datos sobre becas, financiación de proyectos, clasificación de investigadores y categorías profesorales. En general, encontramos desequilibrios de género persistentes en todo el ecosistema de investigación que fueron significativos estadísticamente. Primero, aunque las mujeres constituyen >50% de los egresados de pregrado en ciencias naturales, <40% de los investigadores en este campo son mujeres. Segundo, las mujeres ganan <30% de las convocatorias de investigación, y a su vez, su productividad científica es 2X menor que la de los hombres. Tercero, dada la menor financiación y producción científica de las mujeres, su ascenso a posiciones *senior* en la clasificación de investigadores y a altas categorías profesorales es más lento. En consecuencia, solo ~25% de los investigadores senior y profesores titulares son mujeres. Cuarto, la proporción de mujeres líderes de grupos de investigación y mentoras de jóvenes científicos en Colombia es <30%. Nuestro estudio contribuye a entender las brechas de género en investigación en STEM en Colombia y da información para diseñar iniciativas efectivas que aborden las disparidades de género centrándose en áreas clave de intervención para luego avanzar gradualmente, en lugar de abordar todas las inequidades estructurales de una vez.

Palabras clave: Colombia, mujeres en STEM, efecto tijera, tubería con fugas, inequidad de género

**Introducción**

Las mujeres han ganado mayor participación en investigación en las últimas décadas, especialmente en áreas STEM, donde el número de egresadas es más alto que nunca, incluso alcanzando la paridad mujeres-hombres a nivel de pregrado y maestría [1–3]. Sin embargo, el progreso en la carrera de las mujeres en investigación aún enfrenta muchos desafíos, haciendo que abandonen la academia con mucha más frecuencia que sus colegas hombres [3]. Esta desaparición de mujeres a medida que avanzan en la carrera científica es una tendencia global que suele denominarse “*tubería con fugas*”, que se ve también en una curva en forma de tijera (es decir, el “*efecto tijera*”) [4–7]. En 2019, las mujeres representaban el 44% de los doctorados a nivel mundial, pero solo eran el 29.3% de los investigadores activos, mostrando una preocupante tasa de abandono de las profesiones de investigación [8]. Sin embargo, análisis detallados por país o disciplina individual muestran que las disparidades de género no son las mismas en todas las ciencias o regiones geográficas. En Francia y Alemania, por ejemplo, solo alrededor del 25% de los investigadores en STEM son mujeres, mientras que en Japón esta cifra baja incluso hasta el 15% [9]. Además, un análisis por disciplina realizado en Brasil mostró que mientras el avance de las mujeres investigadoras en las ciencias biológicas y de la salud se ajusta perfectamente a una curva en forma de tijera, dicho patrón no se observa en áreas como la química, geología, ingeniería, matemáticas, física y astronomía, donde las mujeres están consistentemente subrepresentadas en todas las etapas académicas [10].

Además, las mujeres que permanecen en carreras de investigación tienen una menor huella investigativa en comparación con los hombres [11,12]. Muchos estudios han demostrado que existe sesgo en las contribuciones del primer y último autor en revistas de alto impacto, y que incluso al publicar en esas revistas, el trabajo de las mujeres es menos citado que el de los hombres [13]. Las mujeres también tienen una menor participación en redes de colaboración internacional, lo que resulta en menos coautorías y menos producción científica que los hombres [2]. Además, hay evidencia empírica que muestra que la probabilidad de que las mujeres obtengan financiación para investigación es menor, no por la calidad de sus propuestas, sino porque son evaluadas menos favorablemente cuando aparecen como investigadoras principales [14]. Todos estos factores contribuyen a mantener un techo de cristal para las mujeres en la ciencia [14,15], dificultándoles alcanzar posiciones senior, lo que se refleja en que son una proporción menor de los profesores de planta y especialmente de los profesores titulares [16]. Abordar los problemas de brecha de género en la investigación requiere análisis continuos y basados en datos actualizados que ayuden a gobiernos, financiadores e instituciones a implementar iniciativas de equidad basadas en la identificación rigurosa de las barreras que enfrenta una minoría de género. Así se pueden asignar recursos y apoyo que promuevan resultados similares a los de quienes no son minorías de género. Esto es más efectivo que abordar todas las inequidades estructurales de una vez con un enfoque de igualdad que simplemente proporciona los mismos recursos a grupos con características diferentes sin reconocer los desafíos específicos que enfrenta cada uno [17].

Si bien los estudios globales son importantes para alcanzar este objetivo, los estudios regionales y locales tienen más probabilidad de generar acciones institucionales individuales, sin embargo, las estadísticas a nivel nacional sobre las mujeres en ciencia y su uso en la formulación de políticas son aún muy limitadas, especialmente en América Latina [18,19]. En 2016, las mujeres eran el 45.1% de los investigadores en América Latina y el Caribe [8], y las academias de ciencias en la región fueron de las más diversas en cuanto a género, con la mayoría teniendo más del 20% de mujeres como miembros [3]. Estas estadísticas posicionan a América Latina y el Caribe como una de las regiones con mejor desempeño en cuanto a paridad de género a nivel mundial, lo cual es altamente alentador para las investigadoras. Sin embargo, esto no es homogéneo en toda la región, ya que la proporción de mujeres investigadoras varía del 61% al 30% en diferentes países [8]. No obstante, se necesitan análisis locales detallados sobre el progreso en la carrera de las mujeres investigadoras para saber si esa mayor participación de investigadoras es un indicador honesto de mejores condiciones que permiten que las mujeres avancen en sus carreras al mismo ritmo que los hombres. Un estudio reciente en Brasil muestra que, a pesar de una aparente igualdad de género entre los investigadores, la representación de las mujeres en niveles superiores de la escalera académica disminuye (*efecto tijera*) y hay un sesgo de género generalizado en la financiación y la representación de las mujeres en investigación [19]. El más reciente informe de género de Elsevier fue el primero en explorar patrones globales y regionales de participación en la investigación y progreso en la carrera de las mujeres en ciencia, e incluyó a los tres principales países latinoamericanos con mayor producción científica: Brasil, México y Argentina [1,2]. Los hallazgos fueron alentadores. Por ejemplo, Argentina y Brasil fueron los países más cercanos a la paridad de género en la investigación, siendo Argentina el país con la menor diferencia entre hombres y mujeres en producción científica a nivel mundial en 2014-2018. Además, México tuvo el mayor aumento en la proporción de mujeres entre los inventores, aunque ellas sigan siendo minoría. Sin embargo, el informe excluyó países con productividad científica similar a la de Argentina, como Chile y Colombia.

En Colombia, el 90% de las niñas entre 5 y 15 años asisten al colegio y representan ~50% de los estudiantes de secundaria [20]. Las mujeres constituyen el 54% de los estudiantes universitarios, y los egresados de pregrado son un 56% mujeres y un 44% hombres [21]. Las graduadas en campos como salud y bienestar, ciencias sociales, periodismo, administración de empresas, derecho, artes y humanidades son, en su mayoría mujeres, representando entre el 60% y el 70% de los graduados en sus respectivos campos [3]. En contraste, en disciplinas STEM, la proporción de mujeres graduadas es mucho menor, oscilando entre el 23% y el 44% [3], excepto en las ciencias naturales donde las mujeres son el 54.2% de los egresados de pregrado [3]. Esta mayoría femenina persiste en las primeras etapas de la carrera científica. Por ejemplo, en 2019 las mujeres representaron el 54% de los beneficiarios del programa nacional *'Jóvenes Investigadores'*, que promueve el inicio de una carrera científica en personas menores de 28 años en Colombia [22]. Esto sugiere que las ciencias naturales en Colombia han alcanzado la paridad de género, al menos en la base de la escalera científica. Sin embargo, las mujeres solo representan el 34.3% de los investigadores activos en las ciencias naturales [3], lo que muestra que la paridad de género no se aplica a los niveles superiores de dicha escalera. En este contexto, presentamos datos sobre la participación de las mujeres en investigación en Colombia en diferentes etapas de la carrera, analizando las clasificaciones tanto de investigadores como de grupos de investigación y tasas de financiación, con especial énfasis en las ciencias naturales. Enfocamos nuestros análisis en mujeres que se autoidentifican como tal, pero no pudimos abordar otros aspectos de la identidad, como raza, etnia, estatus socioeconómico, orientación sexual o discapacidad, ya que esta información no está disponible para la población objetivo. En la actualidad, los datos disponibles a nivel nacional y organizativo solo nos permiten describir la composición de estudiantes, académicos e investigadores por género, impidiéndonos caracterizar la situación de las personas en intersecciones de identidad, especialmente aquellas en grupos minoritarios.

**Métodos**

***Colecta de datos***

Obtuvimos datos sobre becas y financiación a proyectos de investigación otorgados por el Ministerio de Ciencia Tecnología e Innovación de Colombia (MinCiencias) desde 2012 a través de una solicitud, respondida el 23 de junio de 2022). Para la financiación de proyectos relacionados con las ciencias naturales, solicitamos específicamente información sobre el monto total otorgado y el género del investigador principal (Tabla S1). También solicitamos datos sobre becas doctorales otorgadas para estudios en Colombia y en el extranjero, separando a los beneficiarios por género, y completamos este conjunto de datos con información disponible públicamente en https://minciencias.gov.co/la-ciencia-en-cifras/ para las ciencias naturales. Para las becas posdoctorales en las ciencias naturales descargamos datos disponibles públicamente del sitio web de MinCiencias (https://minciencias.gov.co/la-ciencia-en-cifras/), manteniendo solo la información sobre postdoctorados desarrollados en Colombia.

También descargamos datos públicos de la plataforma nacional de grupos de investigación (GrupLAC) para obtener información sobre la clasificación de los grupos y el género del líder del grupo. Además, obtuvimos la clasificación de cada investigador en las ciencias naturales y su género auto reportado en los años disponibles (2013, 2014, 2015, 2017, 2019 y 2021, https://minciencias.gov.co/la-ciencia-en-cifras/ y https://minciencias.gov.co/ciudadano/datosabiertos). Las clasificaciones para los grupos de investigación van desde A1 hasta D, siendo A1 la clasificación más alta. Las clasificaciones para los investigadores van en el siguiente orden: junior, asociado, senior y emérito. Tanto las clasificaciones de grupos como la de investigadores se asignan principalmente en función de la productividad científica (que incluye artículos y mentoría a estudiantes), y los criterios utilizados por el Ministerio de Ciencia se pueden encontrar en <https://minciencias.gov.co/sites/default/files/upload/convocatoria/anexo_1_-_documento_conceptual_2021.pdf>.

También obtuvimos datos de https://minciencias.gov.co/la-ciencia-en-cifras/ sobre la producción científica en ciencias naturales desglosada por género y año. Es decir, el número de artículos de investigación, libros, capítulos de libros y patentes autoreportados por cada investigador para cada año. Sin embargo, estos datos son acumulativos, por lo que la producción científica de 2013 seguiría contándose en 2021.

Además, recopilamos datos sobre el género del profesorado con doctorado en 86 universidades en Colombia a partir del Sistema Nacional de Información de Educación Superior - SNIES (https://snies.mineducacion.gov.co/, Tabla S2). Dado que estos datos no incluyen la clasificación del profesorado por rango y género, obtuvimos esta información por solicitud a tres universidades importantes en Colombia: (i) Universidad del Tolima, (ii) Universidad de los Andes y (iii) Universidad del Rosario. También incluimos información pública disponible para una cuarta universidad: Universidad Nacional [23]. Restringimos nuestros análisis a estas cuatro instituciones, ya que ninguna otra universidad proporcionó la información solicitada ni tiene estos datos disponibles públicamente. La información para la Universidad del Tolima y la Universidad de los Andes se limitó al año 2023 (Tabla S3), mientras que la Universidad Nacional tenía datos para 2015-2019 y la Universidad del Rosario para 2015-2021 (Tabla S4). En la Universidad del Rosario el rango equivalente a *Profesor Asistente* se llama *Profesor Principal*, pero para fines de comparación, lo renombramos al primero. Debido a limitaciones de disponibilidad, toda esta información es para el profesorado de todas las disciplinas y no solo de las ciencias naturales.

***Análisis estadístico***

Todos los análisis estadísticos se hicieron con el software estadístico R V4.3.1 [24]. Probamos las diferencias significativas en el número de becas doctorales y posdoctorales otorgadas entre géneros y años con pruebas ANOVA dentro de cada categoría (en Colombia o en el extranjero). Las comparaciones entre géneros dentro de cada año se evaluaron con pruebas binomiales ya que no había suficientes datos disponibles para evaluar la interacción entre las dos variables predictoras.

Luego aplicamos pruebas de independencia de *chi*-cuadrado para evaluar si el género está asociado con el rango de un investigador en el sistema científico colombiano (junior, asociado, titular o emérito), o el rango del grupo que lidera un investigador (A1-Reconocido). En ambos casos, utilizamos análisis post hoc para determinar los rangos específicos donde existían diferencias significativas.

**Resultados**

Encontramos que las mujeres están subrepresentadas en el sistema de investigación colombiano en las ciencias naturales, y estas diferencias están presentes desde la formación hasta la consolidación de carrera, siendo más pronunciadas en los rangos superiores. En primer lugar, analizamos datos a nivel de doctorado, reconociendo que hacer un doctorado es el paso más importante para quienes buscan una carrera en investigación científica en ciencias naturales. Encontramos que las becas otorgadas por MinCiencias para programas de doctorado tanto en Colombia como en el extranjero siempre se han dado más a candidatos masculinos que femeninos (Fig. 1). En 2012, por ejemplo, el 40% de las becas para doctorados en Colombia se otorgaron a candidatas femeninas, y este número se ha mantenido constante a lo largo de una década, mostrando poco progreso hacia la paridad de género en la asignación de becas a nivel nacional. De manera similar, para los doctorados en el extranjero, el 34% de las becas se otorgaron a mujeres en 2012, y aunque esta proporción ha mejorado en algunos años, no hay una tendencia sostenida hacia la paridad en la asignación de becas a lo largo del tiempo (Fig. 1). Tanto en programas de doctorado en Colombia como en el extranjero, observamos la proporción más baja de candidatas femeninas que recibieron financiamiento en 2018 (~31.5%) y un aumento reciente en 2021 cuando las beneficiarias femeninas alcanzaron el 44%. Hubo diferencias significativas en el número de becas otorgadas entre géneros y años tanto para becas de doctorado para estudios en el extranjero (p=0.007 para género y p=0.003 para años) como en Colombia (p=0.003 para género y p=3x10^-5^ para años). Encontramos que solo para 2014 los resultados no fueron significativos, y en todos los demás años hubo significativamente menos becas para mujeres en al menos una de las dos categorías (en el extranjero y en Colombia) y para ambas categorías durante cinco años (2012, 2016, 2018, 2019 y 2020, Tabla S5).


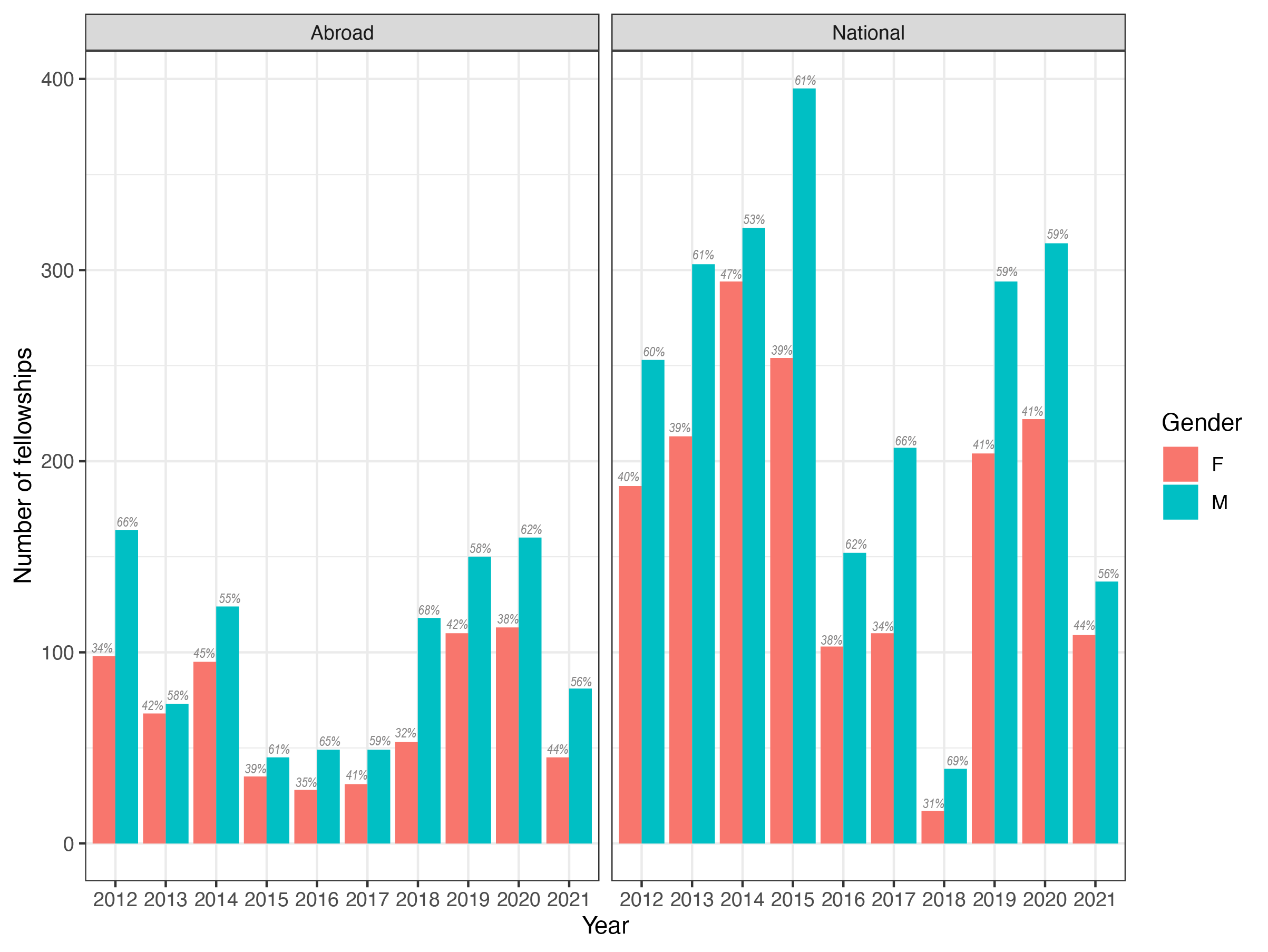


**Figura 1.** **Becas doctorales otorgadas por MinCiencias por año y por género.** El género se muestra en salmón para las mujeres y en azul para los hombres. Los paneles corresponden a becas para doctorados en el exterior (izquierda) y en Colombia (derecha). El tamaño de cada barra indica el número de becas otorgadas por género, y para claridad, el respectivo porcentaje se muestra sobre cada barra.

Después analizamos datos a nivel de posdoctorado, el cual marca la continuación de la carrera científica donde el investigador profundiza en el conocimiento y las habilidades adquiridas durante el doctorado. Desde que iniciaron los fondos de posdoctorado en Colombia en 2017 parece haber paridad de género en la asignación de becas. Así, aunque las mujeres han recibido menos becas a lo largo de los años, las diferencias no son significativas (p= 0.375 para género y p= 0.107 para años). Sin embargo, esto cambió abruptamente en la convocatoria más reciente en 2021, donde la brecha fue la más alta, con un 36% de beneficiarios siendo mujeres y un 64% hombres (Fig. 2).


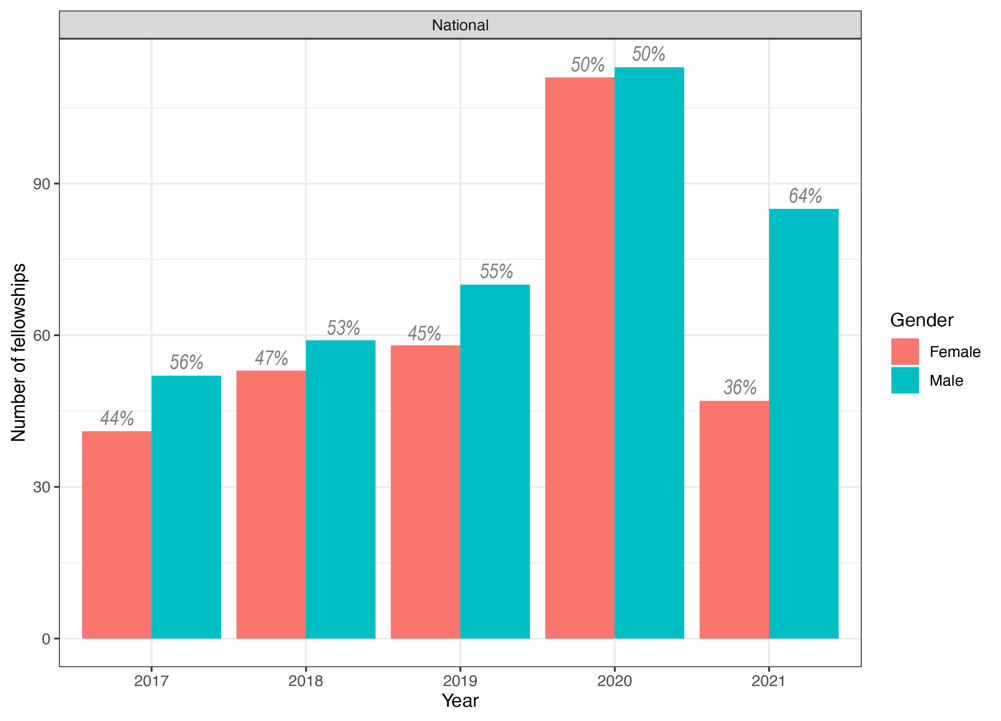


**Figura 2. Becas postdoctorales otorgadas por MinCiencias por año y por género.** El género se muestra en salmón para las mujeres y en azul para los hombres. El tamaño de cada barra indica el número de becas otorgadas por género, y para claridad, el respectivo porcentaje se muestra sobre cada barra. Sólo existen datos a partir del año 2017 ya que antes no existían fondos postdoctorales en Colombia.

En línea con lo anterior, cuando revisamos la clasificación colombiana de investigadores en 2021 en todas las disciplinas, que incluyó a 20,891 investigadores con perfil público (de 21,094 registrados) y 20,295 con información de su género (~97%), encontramos que solo 7,837 o ~39% eran mujeres (Fig. S1). En consecuencia, observamos que el género y el rango del investigador no eran independientes (χ^2^ = 209.35, df = 3, p <2.2x10^-16^), y el análisis post hoc reveló diferencias significativas en todas las categorías excepto en de la de investigador asociado. La representación de las investigadoras fue menor a medida que aumentaba la categoría del investigador, pasando del 42% en la categoría Junior a 29% en la categoría Senior y al 24% en la categoría emérito (Fig. S1). Dentro de las ciencias naturales encontramos que el número de investigadoras reconocidas creció, pasando de 2,080 en 2013 a 4,404 en 2021, es decir, la proporción de mujeres investigadoras aumentó ligeramente del 32% al 36% en esos ocho años (Fig. 3). Para las investigadoras en las ciencias naturales, el género y la categoría del investigador no fueron independientes en ninguno de los años estudiados, y el análisis post hoc mostró diferencias significativas para todas las categorías a lo largo de los años, excepto para la categoría de investigador asociado donde las diferencias no fueron significativas en los años 2017 y 2019 (Fig. 3, Tabla S6). Dentro de cada año, la representación de las investigadoras fue menor a medida que aumentaba la categoría del investigador. En 2013, las investigadoras representaban el 34% de los investigadores en la categoría Junior, el 34% en la categoría Asociado y el 22% en la categoría Senior (Fig. 3). La mejora ha sido modesta en casi una década, y en el último año evaluado, 2021, la participación de las mujeres en el conjunto de investigadores pasó del 39% en la categoría Junior al 35% en la categoría Asociado, al 26% en la categoría Senior y solo al 5% en la categoría emérito (Fig. 3). Por lo tanto, en los últimos ocho años ha habido un ligero aumento en la representación de las investigadoras en las ciencias naturales en las diferentes categorías, similar a su aumento general, pero estos resultados aún están lejos de la paridad y el aumento es sorprendentemente lento.


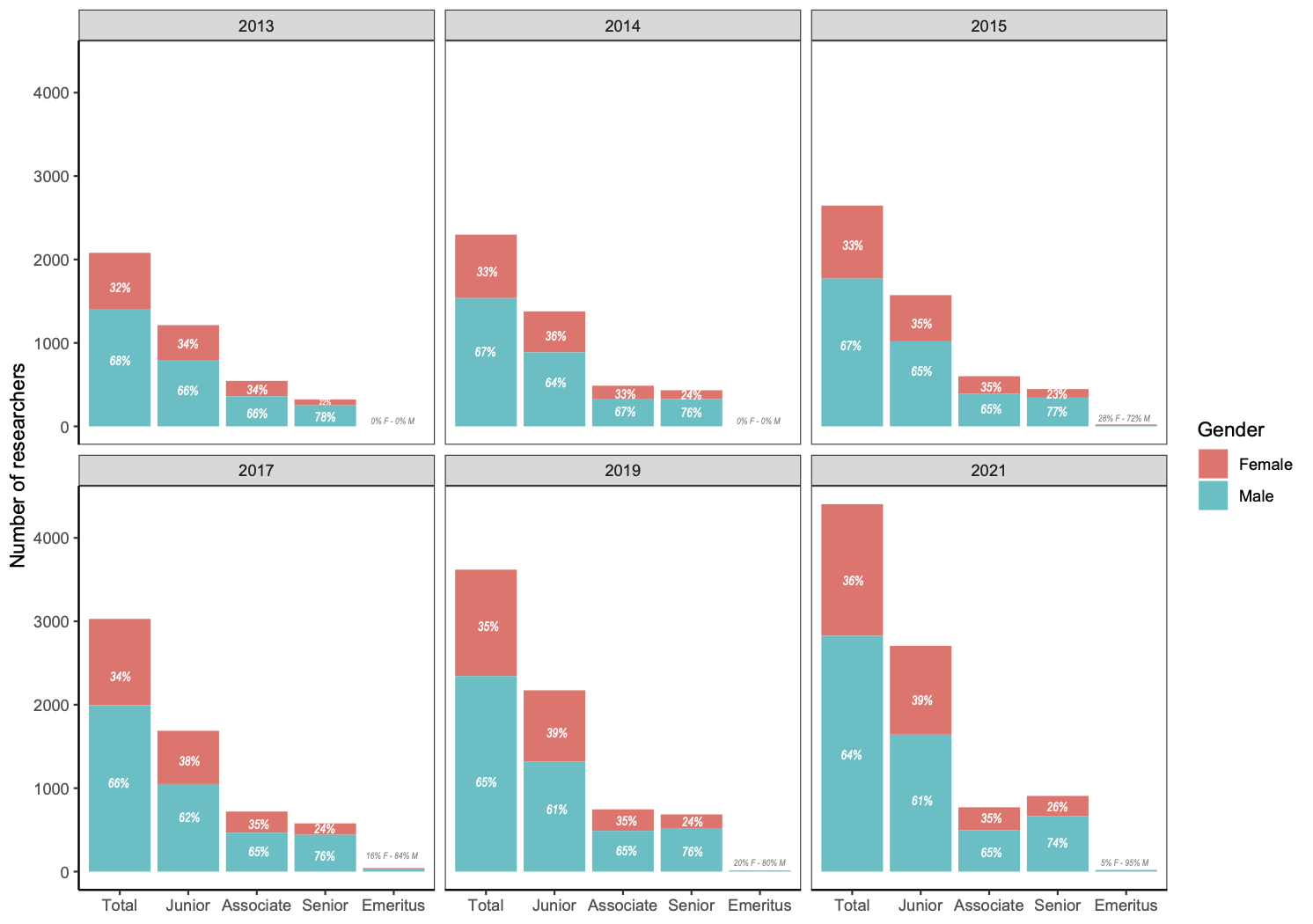


**Figure 3.** **Investigadores reconocidos y su clasificación según el sistema de ciencia colombiano entre 2013 y 2021.** El género de cada investigador fue auto reportado en la base de datos gubernamental y se muestra en salmón para las mujeres y en azul para los hombres. Las categorías van de la más baja a la más alta, y son: “Investigador Junior”, “Investigador Asociado”, “Investigador Senior” e “Investigador Emérito”, mostrados en la figura en su traducción al inglés, así: Junior, Associate, Senior y Emeritus. La barra “Total” muestra el número total de investigadores en todas las categorías. El tamaño de cada barra indica el número de nvestigadores por categoría, y para claridad, el respectivo porcentaje se muestra sobre cada barra.

En consecuencia, aunque en las últimas dos décadas el número total de grupos de investigación reconocidos en ciencias naturales en Colombia ha aumentado, la proporción de mujeres que lideran esos grupos ha permanecido exactamente igual durante casi una década (Fig. 4). Para la mayoría de los años analizados, el género del investigador principal no fue independiente de la categoría del grupo (Tabla S7). Por ejemplo, en 2014, las mujeres lideraban solo el 34% de los grupos de investigación en ciencias naturales, con la mayor proporción de grupos clasificados como A (38%) y C (34%), y solo el 32% clasificados como los mejores (rango A1). La tendencia ha empeorado con el tiempo. En 2021, las mujeres seguían liderando solo el 30% de los grupos de investigación en ciencias naturales, pero que su clasificación disminuyó, con la mayoría de sus grupos clasificados como B (35%) y C (31%), y solo el 23% de ellos clasificados como A1.


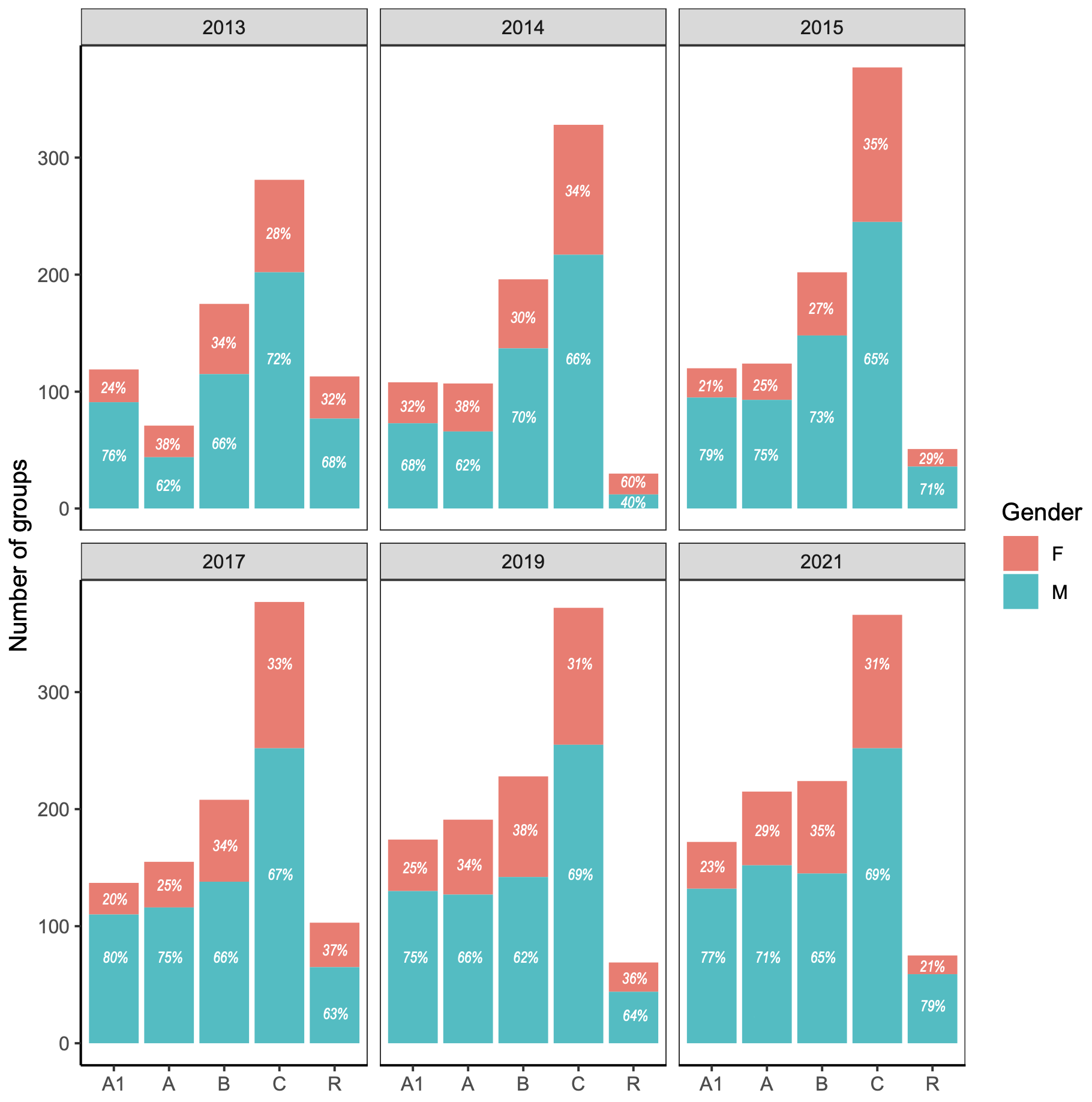


**Figura 4.** **Grupos de investigación** **por categoría y género del líder en los años 2013, 2014, 2015, 2017, 2019 y 2021.** El género del líder del grupo (PI) se asignó con base en el nombre de la persona, y se muestra en salmón para las mujeres y en azul para los hombres. Las categorías van de la más alta A1 a la más baja C. La categoría Reconocido (R) significa que se reconoce la existencia del grupo pero no su clasificación en ninguna categoría. La barra “Total” muestra el número total de investigadores en todas las categorías. El tamaño de cada barra indica el número de grupos por categoría, y para claridad, el respectivo porcentaje se muestra sobre cada barra.

Cuando compilamos datos sobre convocatorias para financiar propuestas de investigación en las ciencias naturales entre 2012 y 2021, encontramos que los montos totales otorgados cada año fueron variables y parecen estar disminuyendo. Desde 2012 hasta 2021 hay un claro sesgo, con más fondos totales otorgados a investigadores principales hombres (Fig. 5), y al observar cada año de forma independiente, encontramos que este sesgo es estadísticamente significativo en cinco de los ocho años analizados (Tabla S8). Sin embargo, al observar proyectos individuales, en promedio, los investigadores reciben la misma financiación por proyecto sin importar el género (Fig. S2), excepto en 2021, cuando hay más variación en los montos individuales otorgados a investigadores principales hombres, los cuales tienden a ser más bajos que los otorgados a líderes de grupo mujeres. Esto concuerda con la proporción de producción científica con autoría de investigadoras mujeres, la cual es menor que la de investigadores hombres. En cuanto a los artículos de investigación, las investigadoras fueron autoras del 27% de los artículos en 2013 y del 29% en 2021 (Tabla S9). Sin embargo, los datos no permiten distinguir la posición del autor (primer y último autor vs. otros) y, por ende, un artículo se contará tantas veces como haya coautores registrados. La menor cantidad de fondos de investigación que reciben las investigadoras también concuerda con la menor mentoría que realizan. Como tal, en 2013, las mujeres dirigieron solo el 22% de las tesis de doctorado y el 30% de las tesis de maestría, y ocho años después, esta proporción sigue sin cambios (Tabla S10).


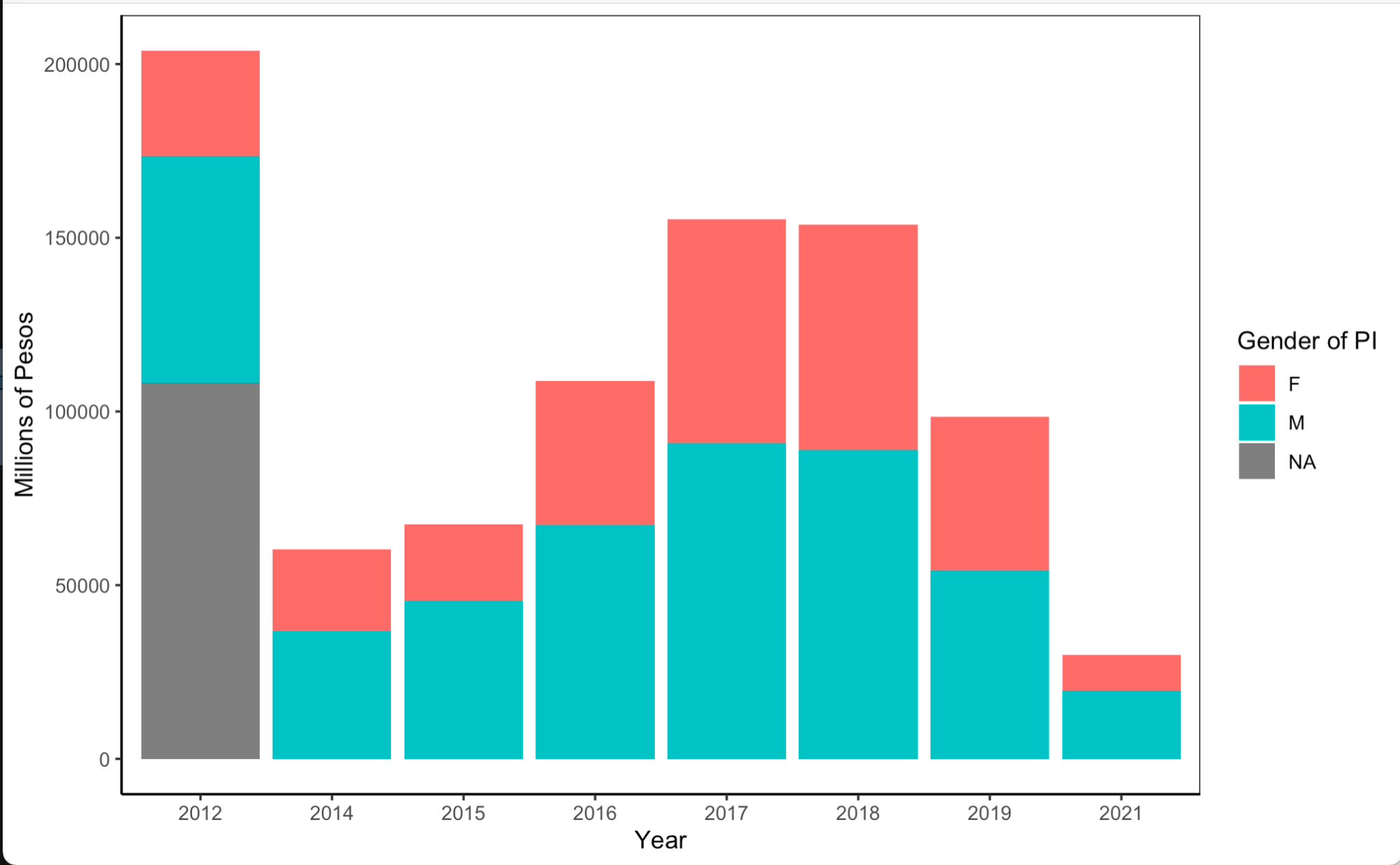


**Figura 5.** **Número total de fondos otorgados por MinCiencias y el género del líder del grupo.** La información de género junto con el monto otorgado fue entregada por MinCiencias. El monto otorgado está en millones de pesos colombianos (1 USD es equivalente a ~4000 COP, según la tasa de cambio de agosto 2023). La lista de convocatorias usadas en el análisis está disponible en el material suplementario, pero en resumen corresponden a las convocatorias en ciencia excluyendo aquellas del programa de regalías. En 2012 casi la mitad de las propuestas no identificaron el género del líder.

Los datos del Sistema Nacional de Información de Educación Superior (SNIES) (https://snies.mineducacion.gov.co/) muestran que el porcentaje de profesoras para todas las áreas de conocimiento tanto en universidades públicas como privadas entre 2015 y 2020 representaba entre el 30% y el 37% del cuerpo docente en todas las disciplinas y categorías profesorales, con una representación femenina ligeramente mayor en universidades privadas que en públicas, constante a lo largo del tiempo (Tabla S2). Hubo un aumento en la representación femenina entre 2015 y 2022, pasando del 31% al 33% en universidades públicas y del 34% al 37% en instituciones privadas (Tabla S2).

Los datos más recientes de instituciones individuales muestran un panorama similar, con las mujeres representando solo ~30% del personal académico en universidades públicas (es decir, Universidad Nacional y Universidad del Tolima) y ~40% en universidades privadas (es decir, Universidad de los Andes y Universidad del Rosario; Fig. 6; Tablas S3 y S4). En todas las instituciones las profesoras están subrepresentadas en todas las categorías profesorales, pero con una representación disminuida en las categorías superiores. Por ejemplo, ~40% de los profesores asistentes en la mayoría de las universidades son mujeres, mientras que, a nivel de asociado, las profesoras son ~35% en universidades privadas y ~30% en las públicas (Fig. 6). Menos del 25% de los profesores titulares son mujeres en las universidades más grandes (es decir, Universidad Nacional y Universidad de los Andes), mientras que en las otras dos instituciones este número estaba apenas por encima del 30% (Fig. 6). Solo en la Universidad del Rosario encontramos más profesoras en dos rangos, pero estos son los rangos más bajos (“Auxiliar”, equivalente a instructores), que están principalmente restringidos a personal sin doctorado, centrados en la enseñanza y con poco tiempo y producción de investigación. También observamos muy poco avance con el tiempo hacia una mayor inclusión de mujeres en los niveles superiores de la escalera académica, al menos para las dos universidades con datos disponibles durante varios años (Figs. S3 y S4). Por ejemplo, en la Universidad del Rosario, la proporción de profesoras aumentó entre 2015 y 2016, pasando del 38% al 45%, pero ha permanecido en su mayoría constante desde entonces, mientras que en la Universidad Nacional no ha habido cambio en la proporción de mujeres/hombres profesores, con los hombres siendo el 70% del cuerpo docente desde 2015 (Figs. S3 y S4).

**Figura 6. Proporción de género en los profesores de planta y su clasificación.** Las mujeres se muestrean en salmón y azul para los hombres. Las categorías, de la más baja a la más alta son: Asistente (“Assistant”), Asociado (“Associate”) y Titular (“Full”). En la Universidad del Rosario el equivalente a profesor asistente se denomina “Principal Professor” pero, para propósitos comparativos, acá lo renombramos. Los datos de la Universidad del Tolima (UTolima) y la Universidad de los Andes (Andes) son para el año 2023. Los datos de la Universidad del Rosario (URosario) son para el 2021. Los datos de la Universidad Nacional (Nacional) son del 2019 y estaban públicamente disponibles [(Bohórquez Montoya et al., 2021)](https://www.zotero.org/google-docs/?kjmgqw). Debido a limitaciones de disponibilidad, los datos corresponden a todas las disciplinas y no solo a las ciencias naturales. El tamaño de la barra indica el número de profesores por categoría, y para claridad se muestra el respectivo porcentaje dentro de cada barra.

**Discusión**

Aunque la investigación en las ciencias naturales en Colombia parece haber alcanzado la paridad de género en las etapas iniciales de la carrera científica (aunque solo considerando una clasificación binaria de género y sin tener en cuenta las interseccionalidades), aquí mostramos que las mujeres están subrepresentadas en el ecosistema de investigación, con diferencias presentes desde la formación hasta la consolidación, pero más pronunciadas en categorías más altas (Fig. 7). En general nuestros hallazgos respaldan la idea de que, al igual que en otros entornos, hay un nivel más allá del cual las investigadoras no pueden avanzar (es decir, existe un techo de cristal).

**
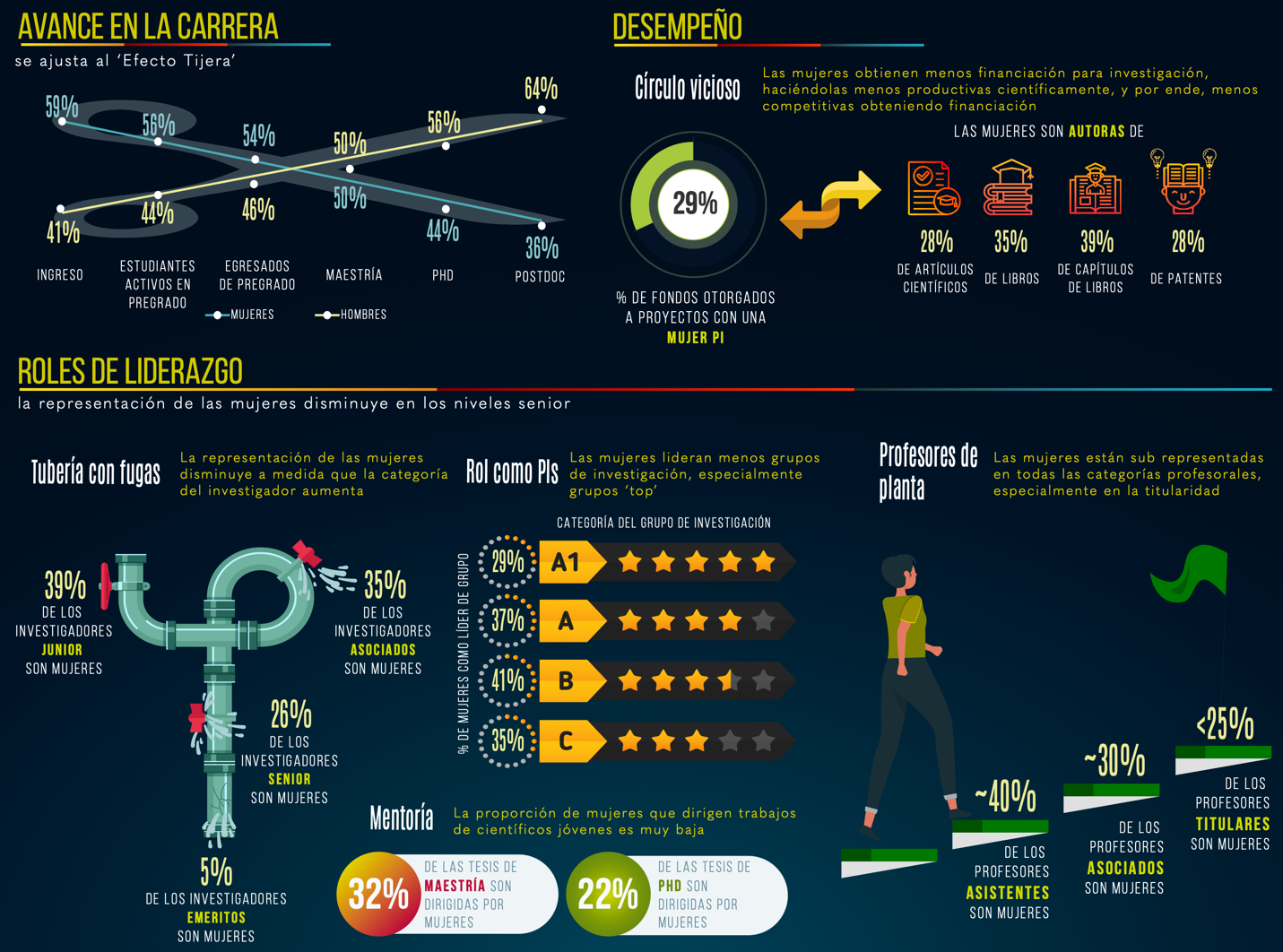
**

**Figura 7. Infografía de la participación de las mujeres en el ecosistema científico Colombiano en ciencias naturales.**

A pesar de que las mujeres son la mayoría de los egresados de pregrado en el país [21] encontramos que, a nivel de doctorado, entre 2015 y 2019 a las mujeres se les otorgó solo el 35% de las becas en todas las disciplinas [22] y el 40% de las becas en ciencias naturales (Fig. 1), lo que implica una disminución de casi 15 puntos porcentuales donde las jóvenes investigadoras desisten de continuar una carrera en ciencia. Luego, a nivel de posdoctorado las mujeres constituyen en promedio el 44% de las beneficiarias de becas posdoctorales tanto en todas las disciplinas como en ciencias naturales [22]. Sin embargo, no hay una tendencia constante hacia la paridad de género, sino que, por el contrario, en la convocatoria más reciente de 2021 solo el 36% de las becas posdoctorales en ciencias naturales se otorgaron a mujeres (Fig. 2).

Si bien el número de investigadores, tanto hombres como mujeres, ha aumentado en la última década en Colombia [22], encontramos que las mujeres representan menos del 40% de los investigadores en ciencias naturales, y, en consecuencia, solo lideran el 30% de los grupos de investigación en el campo. Al analizar los datos con más detalle, surgió una realidad aún más desigual. Las mujeres están representadas de manera desproporcionada en los niveles más bajos de la escalera de investigación, siendo en su mayoría clasificadas como investigadoras junior que lideran grupos en los rangos inferiores del sistema nacional de clasificación (principalmente grupos B y C). Esto va más allá de ser solo un problema estadístico y tiene implicaciones directas en las oportunidades de financiación, ya que todas las convocatorias nacionales otorgan puntos adicionales a las propuestas presentadas por grupos de investigación en los rangos superiores (A o A1) y por ser investigadores senior. No resulta sorprendente que las investigadoras colombianas tengan menos éxito en obtener financiación para proyectos en comparación con sus colegas hombres, en línea con tendencias globales [12], lo que se traduce en que las mujeres desarrollan menos investigación y producen menos resultados. Así, las investigadoras en ciencias naturales son autoras en aproximadamente el 30% de las publicaciones informadas a MinCiencias, lo que está ~10 puntos porcentuales por debajo de las estadísticas a nivel global y podría ser peor ya que las mujeres tienen menos probabilidades de recibir crédito como autoras por su trabajo [12], y los hombres tienden a invitar a más hombres como coautores [25], por lo que un solo artículo puede contarse muchas veces para los investigadores hombres inflando su participación. Esto se ajusta al patrón nacional en todas las disciplinas, donde solo el 28% de los artículos de investigación tienen a mujeres como autoras y la mayoría (41%) de los resultados de investigación producidos por mujeres están relacionados con actividades de divulgación y no generación de conocimiento [22]. Este círculo vicioso lleva a las investigadoras en Colombia a tener menos oportunidades de fortalecer su perfil científico, lo que se traduce en que avanzan en la escalera científica a un ritmo mucho más lento que los hombres [17,26]. En consecuencia, a lo largo de ocho años, solo evidenciamos un ligero aumento (~4 puntos porcentuales) en la representación de las mujeres en el sistema nacional de investigación como líderes de grupos de investigación e investigadoras reconocidas.

Las desigualdades de género que observamos se ajustan a un patrón en forma de tijera (Fig. 7) y son el resultado de la expulsión temprana en la carrera de las mujeres [27]. Las causas son diversas y se han discutido profundamente en la literatura, donde generalmente el cuidado de los hijos y otras responsabilidades de cuidado aparecen como los principales factores explicativos [27–29]. Si bien esto puede ser parcialmente cierto, no explica por qué este sesgo afecta a las mujeres sin hijos o responsabilidades de cuidado, y, además, no explica por qué la brecha de género se acentúa bruscamente cuando las mujeres ya tienen puestos académicos (es decir, no abandonaron su carrera científica). De hecho, el avance de las investigadoras en Colombia hacia categorías académicas y científicas más altas es un tubería con fugas donde las mujeres disminuyen en puntos específicos (Fig. 7), un patrón también observado en América del Norte y Europa [30]. En los Estados Unidos, por ejemplo, las mujeres se convierten en investigadoras principales a un ritmo un 20% menor que los hombres, principalmente debido a que tienen menos publicaciones y citaciones por publicación, lo que explica aproximadamente el 60% de la brecha de género en la adjudicación de financiación [31]. Al igual que en esas regiones, en Colombia, el avance de los investigadores hacia categorías más altas depende en gran medida de la producción científica, la obtención de financiación y la mentoría de estudiantes, y es probable que las mujeres progresen más lentamente que los hombres debido a su menor desempeño en estos indicadores (Fig. 7). Esto sugiere que las mujeres enfrentan barreras de género que limitan sus carreras en un país donde realizar investigación es un desafío en sí mismo: la inversión del gobierno en I+D es demasiado baja (<0.25% del PIB en la última década [3]) y el conflicto armado que el país ha enfrentado durante más de 50 años ha profundizado estas barreras, afectando especialmente a las mujeres [32].

Una posible causa de este patrón es el sesgo de género, es decir, un prejuicio explícito o implícito basado en el género que afecta a múltiples instancias del desempeño de las mujeres [30]. Primero, los sesgos de género en las publicaciones han sido confirmados con investigación experimental y observacional, incluyendo la aleatorización de nombres en CV y resúmenes enviados para evaluación, y la evaluación de números de citas y reconocimiento de nombres [33,34]. Segundo, el tiempo que las mujeres tienen disponible para la investigación se ve influenciado por los sesgos de género. Mientras que generalmente las mujeres tienen menos tiempo para la investigación debido a su carga doméstica, una gran proporción de la carga administrativa y docente en entornos académicos se asigna a las mujeres [35], impactando directamente en su productividad, satisfacción laboral y niveles de estrés [36–38]. Tercero, un sesgo de género adicional proviene de que las mujeres investigadoras juniors tienen menos modelos a seguir femeninos que puedan actuar como mentoras y apoyarlas en la comprensión de los procesos de publicación y solicitud de financiación, así como en la presentación y relacionamiento en una red de colaboradores [31].

Nuestros datos muestran que, a pesar del aumento general en la participación de las mujeres en la investigación, y en particular en las ciencias naturales en Colombia, simplemente aumentar el número de investigadoras no es suficiente para garantizar la paridad, lo cual es una observación consistente en diferentes países y disciplinas [29]. En Colombia las políticas locales que buscan mejorar la participación de las mujeres en la ciencia han seguido el enfoque habitual de motivar la matrícula de mujeres en programas científicos, y al menos en los niveles de pregrado y jóvenes investigadoras, ha funcionado [22]. Sin embargo, el país necesita diseñar e implementar políticas nuevas y más asertivas con énfasis especial en: (i) identificar y abordar las causas de deserción de las jóvenes investigadoras, (ii) promover la inscripción de investigadoras con doctorado reciente en el ecosistema científico, y (iii) mitigar las bajas tasas de retención y avance de las investigadoras en el sistema científico y académico. Estudios han demostrado que centrarse solo en jóvenes científicas no es suficiente para reducir el desequilibrio de género en la ciencia [29] y, por lo tanto, abordar los dos últimos problemas es imperativo. Las convocatorias de financiación para becas postdoctorales en 2023 de MinCiencias están dando preferencia (puntos extras) por primera vez a las postulantes mujeres, así como a grupos minoritarios, lo que se espera que motive la inserción de doctoras junior en el sistema de investigación colombiano. Los resultados y el impacto de estas nuevas medidas aún deben ser evaluados ya que los fondos aún no se han asignado. En cuanto a obtener puestos de profesorado a nivel de ingreso, las convocatorias de contratación para posiciones de tiempo completo en universidades públicas y privadas, quienes hacen la mayoría de la investigación en Colombia, generalmente no toman ninguna medida para fomentar las aplicaciones de mujeres, lo que dificulta lograr cambios hacia la paridad de género en los rangos de investigadores y profesores. La única excepción es la Universidad Industrial de Santander, que en noviembre de 2023 lanzó la primera convocatoria de contratación con enfoque de género [39], convirtiéndose en la primera institución en Colombia en implementar medidas específicas para promover la equidad de género entre su personal académico. Solo una de las cuatro universidades estudiadas tiende hacia la paridad de género en la categoría de entrada para profesores de tiempo completo (Universidad de los Andes, Fig. 6), y solo en esta institución se podría esperar la paridad de género en las categorías más altas a medio y largo plazo (>5 años). Sin embargo, esto difícilmente se espera en las otras instituciones. La persistencia prevista de estas desigualdades en categorías más altas es motivo de especial preocupación porque los investigadores altamente clasificados sirven como el principal modelo a seguir para las nuevas generaciones de científicos, y la subrepresentación de las mujeres en esos puestos perpetúa el estereotipo de que solo los hombres pueden ser investigadores exitosos. De hecho, un estudio reciente mostró que los estudiantes de pregrado en Colombia perciben a sus profesores hombres como 'cracks' mientras que sus profesoras mujeres fueron percibidas como 'amables' [40], subrayando la importancia que juegan los modelos a seguir y cuán crucial es hacer visible el trabajo y los logros de las investigadoras.

Desde becarias hasta profesoras titulares las muejres representan un tercio del total de los investigadores. Reconocemos que existen sesgos más allá del género que afectan a las investigadores del sur global, hablantes no nativos de inglés, otros grupos minoritarios como personas negras, indígenas y LGBTIQ+, y diversas interseccionalidades, entre otros [45]. Sin embargo, en este trabajo nos centramos en el género ya que es una categoría auto-informada por los investigadores. En la actualidad no hay información formal sobre otros problemas que enfrentan las mujeres en la academia, como el acoso, problemas de salud mental, discriminación racial, entre otros. Esto nos impide analizar cómo estos también afectan su progreso en la escalera académica. Por lo tanto, es necesario recopilar datos sobre otras poblaciones minorizadas, incluidas la raza, el estatus socioeconómico y la identidad de género, para identificar y actuar contra dichos sesgos [46]. Aplaudimos al Ministerio por haber iniciado un esfuerzo para recopilar estos datos e incluir consideraciones de diversidad en sus convocatorias más recientes para becas de formación.

**Agradecimientos**

Las autoras agradecen al Ministerio de Ciencia, Tecnología e Innovación por proporcionar los datos solicitados sobre becas, financiación de proyectos e investigadores. También queremos agradecer a la Universidad del Tolima, la Universidad de los Andes y la Universidad del Rosario por proporcionar datos sobre el cuerpo docente en sus instituciones. Las autoras agradecen a Valeria Ramírez-Castañeda y a dos revisores anónimos por comentarios que ayudaron a mejorar significtivamente este manuscrito.

**REFERENCES**

1. De Kleijn, M, Jayabalasingham, B, Falk-Krzesinski, HJ, Collins, T, Kuiper-Hoyng, L, Cingolani, I, et al. The Researcher Journey Through a Gender Lens: An Examination of Research Participation, Career Progression and Perceptions Across the Globe. 2020. Available: www.elsevier.com/gender-report

2. Elsevier. Gender in the global research landscape: analysis of research performance through a gender lens across 20 years, 12 geographies, and 27 subject areas. 2017.

3. UNESCO. UNESCO Science Report 2021: The Race Against Time for Smarter Development. United Nations; 2021. doi:10.18356/9789210058575

4. Martinez ED, Botos J, Dohoney KM, Geiman TM, Kolla SS, Olivera A, et al. Falling off the academic bandwagon. EMBO Rep. 2007;8: 977–981. doi:10.1038/sj.embor.7401110

5. OECD. Women in Scientific Careers: Unleashing the Potential. Paris: Organisation for Economic Co-operation and Development; 2006. Available: https://www.oecd-ilibrary.org/employment/women-in-scientific-careers_9789264025387-en

6. Ysseldyk R, Greenaway KH, Hassinger E, Zutrauen S, Lintz J, Bhatia MP, et al. A Leak in the Academic Pipeline: Identity and Health Among Postdoctoral Women. Front Psychol. 2019;10. Available: https://www.frontiersin.org/articles/10.3389/fpsyg.2019.01297

7. Shaik F, Fusulier B. Academic Careers and Gender Inequality: Leaky Pipeline and Interrelated Phenomena in Seven European Countries. 2015. Available: https://dial.uclouvain.be/pr/boreal/object/boreal:168170

8. UNESCO Institute for Statistics. Women in science - UNESCO Digital Library. Montreal, Canada: UNESCO; 2019. Report No.: UIS fact sheet, no. 55. Available: https://unesdoc.unesco.org/ark:/48223/pf0000370742?posInSet=1&queryId=5a0da293-19ca-4f4a-868b-420103351fbc

9. Celebrating women in science. Nat Cell Biol. 2018;20: 993–993. doi:10.1038/s41556-018-0190-4

10. Bezerra G, Lazzaro D, Daniela Lazzaro, Peixoto Aruquia, Lopes MM, Andrade CH, et al. Female Researchers in Science in Brazil: the Scissors Effect. 2018. Available: https://www.academia.edu/79372525/Female_Researchers_in_Science_in_Brazil_the_Scissors_Effect

11. Ioannidis JPA, Boyack KW, Collins TA, Baas J. Gender imbalances among top-cited scientists across scientific disciplines over time through the analysis of nearly 5.8 million authors. PLOS Biol. 2023;21: e3002385. doi:10.1371/journal.pbio.3002385

12. Ross MB, Glennon BM, Murciano-Goroff R, Berkes EG, Weinberg BA, Lane JI. Women are credited less in science than men. Nature. 2022;608: 135–145. doi:10.1038/s41586-022-04966-w

13. Bendels MHK, Müller R, Brueggmann D, Groneberg DA. Gender disparities in high-quality research revealed by Nature Index journals. PLOS ONE. 2018;13: e0189136. doi:10.1371/journal.pone.0189136

14. Witteman HO, Hendricks M, Straus S, Tannenbaum C. Are gender gaps due to evaluations of the applicant or the science? A natural experiment at a national funding agency. The Lancet. 2019;393: 531–540. doi:10.1016/S0140-6736(18)32611-4

15. Bruckmüller S, Ryan MK, Rink F, Haslam SA. Beyond the Glass Ceiling: The Glass Cliff and Its Lessons for Organizational Policy. Soc Issues Policy Rev. 2014;8: 202–232. doi:10.1111/sipr.12006

16. Danell R, Hjerm M. Career prospects for female university researchers have not improved. Scientometrics. 2013;94: 999–1006. doi:10.1007/s11192-012-0840-4

17. Jebsen JM, Nicoll Baines K, Oliver RA, Jayasinghe I. Dismantling barriers faced by women in STEM. Nat Chem. 2022;14: 1203–1206. doi:10.1038/s41557-022-01072-2

18. Areas R, Abreu ARP, Santana AE, Barbosa MC, Nobre C. Gender and the scissors graph of Brazilian science: from equality to invisibility. Open Science Framework; 2020 Jun. doi:10.31219/osf.io/m6eb4

19. Zandonà E. Female ecologists are falling from the academic ladder: A call for action. Perspect Ecol Conserv. 2022;20: 294–299. doi:10.1016/j.pecon.2022.04.001

20. Daza-Caicedo S, Farías D, Ariza V. El eterno mito de la igualdad: el caso de las mujeres en el SNCTI. Análisis de Indicadores de Ciencia y Tecnología. Bogota, Colombia: Editorial Universidad del Rosario; 2016. pp. 279–333.

21. ONU Mujeres, DANE, CPEM. Mujeres y hombres: brechas de género en Colombia. 2020. Available: https://colombia.unwomen.org/es/biblioteca/publicaciones/2020/11/mujeres-y-hombres-brechas-de-genero-en-colombia

22. Ardila DS, Pérez AM, Dávila MAM, Holguín HM, Aguilar SR, García M, et al. Indicadores de ciencia, tecnología e innovación. Colombia 2020. Observatorio Colombiano de Ciencia y Tecnología - OCyT. 2020.

23. Bohórquez Montoya JP, Marín Guzmán JI, Ramos Urrego LL. Estudio sociodemográfico caracterización del personal docente y administrativo de planta de la Universidad Nacional de Colombia desde una perspectiva de género e interseccional 2015-2019. 2021.

24. R Core Team. R: A language and environment for statistical computing. Vienna, Austria: R Foundation for Statistical Computing; 2023. Available: https://www.r-project.org/

25. Frances DN, Fitzpatrick CR, Koprivnikar J, McCauley SJ. Effects of Inferred Gender on Patterns of Co-Authorship in Ecology and Evolutionary Biology Publications. Bull Ecol Soc Am. 2020;101: e01705. doi:10.1002/bes2.1705

26. O’Connell C, McKinnon M. Perceptions of Barriers to Career Progression for Academic Women in STEM. Societies. 2021;11: 27. doi:10.3390/soc11020027

27. Rivera LA. When Two Bodies Are (Not) a Problem: Gender and Relationship Status Discrimination in Academic Hiring. Am Sociol Rev. 2017;82: 1111–1138. doi:10.1177/0003122417739294

28. Ceci SJ, Williams WM. Understanding current causes of women’s underrepresentation in science. Proc Natl Acad Sci. 2011;108: 3157–3162. doi:10.1073/pnas.1014871108

29. Huang J, Gates AJ, Sinatra R, Barabási A-L. Historical comparison of gender inequality in scientific careers across countries and disciplines. Proc Natl Acad Sci. 2020;117: 4609–4616. doi:10.1073/pnas.1914221117

30. Llorens A, Tzovara A, Bellier L, Bhaya-Grossman I, Bidet-Caulet A, Chang WK, et al. Gender bias in academia: A lifetime problem that needs solutions. Neuron. 2021;109: 2047–2074. doi:10.1016/j.neuron.2021.06.002

31. Lerchenmueller MJ, Sorenson O. The gender gap in early career transitions in the life sciences. Res Policy. 2018;47: 1007–1017. doi:10.1016/j.respol.2018.02.009

32. Franco-Orozco CM, Franco-Orozco B. Women in Academia and Research: An Overview of the Challenges Toward Gender Equality in Colombia and How to Move Forward. Front Astron Space Sci. 2018;5. doi:10.3389/fspas.2018.00024

33. González MJ, Cortina C, Rodríguez J. The Role of Gender Stereotypes in Hiring: A Field Experiment. Eur Sociol Rev. 2019;35: 187–204. doi:10.1093/esr/jcy055

34. Knobloch-Westerwick S, Glynn CJ, Huge M. The Matilda Effect in Science Communication: An Experiment on Gender Bias in Publication Quality Perceptions and Collaboration Interest. Sci Commun. 2013;35: 603–625. doi:10.1177/1075547012472684

35. Guarino CM, Borden VMH. Faculty Service Loads and Gender: Are Women Taking Care of the Academic Family? Res High Educ. 2017;58: 672–694. doi:10.1007/s11162-017-9454-2

36. Pedersen DE, Minnotte KL. University Service Work in STEM Departments: Gender, Perceived Injustice, and Consequences for Faculty. Sociol Focus. 2018;51: 217–237. doi:10.1080/00380237.2018.1393607

37. Minnotte KL, Pedersen DE. Turnover Intentions in the STEM Fields: The Role of Departmental Factors. Innov High Educ. 2021;46: 77–93. doi:10.1007/s10755-020-09524-8

38. Jebsen JM, Abbott C, Oliver R, Ochu E, Jayasinghe I, Gauchotte-Lindsay C. Review of barriers women face in research funding processes in the UK. Psychol Women Equal Sect Rev. 2020;3: 3–14. doi:10.53841/bpspowe.2020.3.1-2.3

39. Porras Diaz H. Acuerdo No. 376 de 2023. Universidad Industrial de Santander; 2023. Available: https://comunicaciones.uis.edu.co/wp-content/uploads/2023/11/Acuerdo-376-de-2023.pdf

40. Urdaneta Andrade N. ¿Hombres “cracks” y mujeres “amables”? Sesgos de género en encuestas de profesores. 2021. Available: http://hdl.handle.net/1992/51803

41. Meneghini R, Packer AL, Nassi-Calò L. Articles by Latin American Authors in Prestigious Journals Have Fewer Citations. PLOS ONE. 2008;3: e3804. doi:10.1371/journal.pone.0003804

42. Amano T, González-Varo JP, Sutherland WJ. Languages Are Still a Major Barrier to Global Science. PLOS Biol. 2016;14: e2000933. doi:10.1371/journal.pbio.2000933

43. Amano T, Ramírez-Castañeda V, Berdejo-Espinola V, Borokini I, Chowdhury S, Golivets M, et al. The cost of being a non-native English speaker in science. Biodiversity; 2022 Nov. doi:10.32942/X29G6H

44. Ramírez-Castañeda V. Disadvantages in preparing and publishing scientific papers caused by the dominance of the English language in science: The case of Colombian researchers in biological sciences. PLOS ONE. 2020;15: e0238372. doi:10.1371/journal.pone.0238372

45. Diele-Viegas LM, Sales LP, Slobodian V, Virginio F, de Araújo Sousa S, Pareja-Mejía D, et al. Productivity in academia: When the rules determine the losers. Front Ecol Evol. 2022;10. Available: https://www.frontiersin.org/articles/10.3389/fevo.2022.1021812

46. McNair TB, Bensimon EM, Malcom-Piqueux L. From Equity Talk to Equity Walk. John Wiley & Sons, Ltd; 2020. doi:10.1002/9781119428725.ch1

47. Buckles K. Fixing the Leaky Pipeline: Strategies for Making Economics Work for Women at Every Stage. J Econ Perspect. 2019;33: 43–60. doi:10.1257/jep.33.1.43

48. Devine PG, Forscher PS, Cox WTL, Kaatz A, Sheridan J, Carnes M. A Gender Bias Habit-Breaking Intervention Led to Increased Hiring of Female Faculty in STEMM Departments. J Exp Soc Psychol. 2017;73: 211–215. doi:10.1016/j.jesp.2017.07.002

49. O’Meara K, Culpepper D, Templeton LL. Nudging Toward Diversity: Applying Behavioral Design to Faculty Hiring. Rev Educ Res. 2020;90: 311–348. doi:10.3102/0034654320914742

**Tabla 1. Algunas sugerencias para mitigar el sesgo de género en la comunidad investigativa Colombiana.** Presentamos algunas sugerencias de la literatura sobre acciones que podrían ayudar a mitigar algunos de los sesgos que observamos en la comunidad investigativa. No es una lista larga ni un conjunto de acciones mágicas.

| **Justificación** | **Acción** | **Referencias** |
| --- | --- | --- |
| Las mujeres en la categoría de profesor asistente enfrentan la presión del proceso de permanencia o promoción con compromisos familiares crecientes y sin estructuras formales de mentoría. | Crear espacios de mentoría específicos dentro de sociedades científicas o universidades centrados en proporcionar información y entrenamiento sobre cómo estructurar una postulación a promoción profesoral. Esto aumentaría la confianza en el proceso de promoción y reduciría el impacto de un acceso limitado a otras redes. | [47] |
| Las mujeres hacen más trabajo de servicio administrativo interno, el cual es menos valorado en procesos de promoción profesoral. | Desarrollar expectativas administrativas claras a través de planes de servicio administrativo para todos los profesores (hombres y mujeres), de forma en que sea más fácil detectar asignaciones administrativas asimétricas. | [47] |
| Los workshops en “Intervenciones para romper hábitos de sesgo de género” en Universidades focales han mostrado un aumento en la probabilidad de hacer ofertas laborales a mujeres. | Promover la participación de profesores en intervenciones para romper hábitos de sesgo de género diseñadas para generar consciencia, analizar consecuencias y proponer estrategias del sesgo de género en ambientes académicos e investigativos. | [48,49] |
| Los mecanismos actuales de asignación de fondos dependen de los logros pasados como indicadores confiables para evaluar el potencial de los postulantes, lo que dificulta que los jóvenes investigadores que ingresan al sistema compitan por fondos que les permitan iniciar su programa de investigación independiente, especialmente en ausencia de fondos de arranque (que suele ser el caso en Colombia). | Crear un “Fondo Gubernamental Básico de Investigación” dirigido a profesores asistentes recientemente contratados y que les permita tener acceso a un monto básico anual. | [17] |
| Las universidades no tienen incentivos reales para promover la paridad de género | La acreditación de alta calidad de Instituciones de Educación Superior debería dar puntaje al compromiso demostrado de una institución para alcanzar la paridad de género en su cuerpo académico. | [17] |
| Las mujeres tienen más responsabilidades de cuidado (maternidad, cuidado de familiares mayores, etc.) | Considerar las responsabilidades de cuidado en los procesos de promoción profesoral o de investigación y evaluaciones de proyectos (aumentando la ventana de observación para estos casos), y brindar infraestructura de guardería. | [50,51] |

**Tablas y figuras suplementarias (solo leyendas)**

**TABLAS**

**Tabla S1.** **Convocatorias incluidas en la información solicitada a MinCiencias**. La solicitud se hizo incluyendo el nombre, número y año de las convocatorias de interés. La solicitud de información se envió en Junio 23, 2022.

**Tabla S2.** **Género de profesores de planta con título doctoral en 86 universidades en Colombia divididos por tipo de universidad: pública o privada entre 2015 y 2021**. Los datos se obtuvieron del Sistema Nacional de Información en Educación Superior - SNIES (<https://snies.mineducacion.gov.co/>)

**Tabla S3.** **Porcentaje de profesoras mujeres en la Universidad de los Andes (privada) y la Universidad del Tolima (pública) en las diferentes categorías profesorales.** Datos obtenidos por solicitud.

**Tabla S4. Porcentaje de profesoras mujeres en la Universidad del Rosario (privada) en las diferentes categorías profesorales entre el 2015 y 2020.** Datos obtenidos por solicitud. Las categorías van de la más baja a la más alta en el siguiente orden: “Auxiliar”, “Asistente”, “Principal”, “Asociado”, y “Titular”.

**Tabla S5.** **Prueba binomial para las becas doctorales otorgadas a mujeres para estudiar en el exterior o Colombia.** Los años significativos (con valor p <0.05) para estudios en el exterior se señalan con un asterisco negro (*), y para estudios en Colombia con un asterisco gris (*). Los años significativos para estudios nacionales y extranjeros se marcan en negrilla.

**Tabla S6. Resultados de prueba de independencia χ^2^ entre categoría del investigador y género.** Los años significativos (con valor p <0.05) se marcan con un asterisco (*).

**Tabla S7. Resultados de prueba de independencia χ^2^ entre categoría del grupo de investigación y género del líder del grupo.** Los años significativos (con valor p <0.05) se marcan con un asterisco (*).

**Tabla S8.** **Prueba binomial para los proyectos de investigación financiados a mujeres en el periodos 2012-2021.** Se usó únicamente el número de proyectos financiados y no el monto otorgado. Los años con menos del 50% de los proyectos financiados a mujeres (con valor p <0.05) se marcan con un asterisco (*).

**Table S9.** **Productividad académica de investigadores colombianos entre 2013 y 2021.** Datos separados por género y tipo de producto.

**Tabla S10. Supervisión de tesis (mentoría) a nivel de maestría y doctorado hecha por investigadores colombianos entre 2013 y 2021**. Datos separados por género del supervisor.

**FIGURES**

**Figura S1. Número de investigadores reconocidos y su clasificación en la última convocatoria de reconocimiento de MinCiencias en 2021**. El género de los investigadores se auto reporta en la base de datos del gobierno y se muestra aquí como femenino en color salmón y masculino en azul. Las categorías, de menor a mayor, son: investigador junior, investigador asociado, investigador senior e investigador emérito. La barra “Total” muestra el número total de investigadores en todas las categorías.

**Figura S2.** **Fondos otorgados por MinCiencias por año y proyecto individual según el género del líder del grupo entre 2012 y 2021**. MinCiencias dio la información de género del investigador principal junto con el monto financiado. Los montos otorgados están en millones de pesos colombianos (1 USD ~ 4100 COP, aunque la tasa de cambio es variable). La lista de convocatorias de proyectos utilizadas para los análisis está disponible en el material suplementario, pero, en resumen, corresponden a convocatorias en ciencia y excluyen los programas especiales de regalías.

**Figura S3. Proporción de género de profesores de tiempo completo y sus categorías en la Universidad del Rosario entre 2015 y 2021.** Femenino en color salmón y masculino en azul. Para fines de equivalencia y claridad, las categorías internas se renombraron para ser equivalentes al estándar internacional. Las categorías de menor a mayor son: Auxiliar 1 (institucionalmente llamado “Auxiliar”), Auxiliar 2 (institucionalmente llamado “Asistente”), Asistente (institucionalmente llamado “Principal”), Asociado (institucionalmente llamado “Asociado”) y Titular (institucionalmente llamado "Titular"). Auxiliar 1 y 2 son equivalentes a instructores en la clasificación internacional. Debido a limitaciones de disponibilidad, los datos son para todas las disciplinas y no solo para las ciencias naturales.

**Figura S4. Proporción de género de profesores de tiempo completo y sus categorías en la Universidad Nacional entre 2015 y 2019.** Femenino en color salmón y masculino en azul. Las categorías de menor a mayor son: Auxiliar (institucionalmente llamado “Auxiliar”), Asistente (institucionalmente llamado “Asistente”), Asociado (institucionalmente llamado “Asociado”) y Titular (institucionalmente llamado “Titular”). Auxiliar es equivalente a instructores en la clasificación internacional. Los datos fueron publicados originalmente en Bohórquez Montoya et al., 2021, pero debido a limitaciones de disponibilidad, son para todas las disciplinas y no solo para las ciencias naturales.
